# Dentate Granule Cells Recruited in the Home Environment Display Distinctive Properties

**DOI:** 10.3389/fncel.2020.609123

**Published:** 2021-01-15

**Authors:** Claire Pléau, Angélique Peret, Edouard Pearlstein, Thomas Scalfati, Alexandre Vigier, Geoffrey Marti, François J. Michel, Thomas Marissal, Valérie Crépel

**Affiliations:** ^1^INMED, INSERM UMR1249, Aix-Marseille University, Marseille, France; ^2^ISM, Aix-Marseille University, CNRS, Marseille, France

**Keywords:** hippocampus, dentate granule cell, excitability, shunting inhibition, home environment

## Abstract

The dentate granule cells (DGCs) play a crucial role in learning and memory. Many studies have described the role and physiological properties of these sparsely active neurons using different behavioral contexts. However, the morpho-functional features of DGCs recruited in mice maintained in their home cage (without training), considered as a baseline condition, have not yet been established. Using fosGFP transgenic mice, we observed *ex vivo* that DGCs recruited in animals maintained in the home cage condition are mature neurons that display a longer dendritic tree and lower excitability compared with non-activated cells. The higher GABA_A_ receptor-mediated shunting inhibition contributes to the lower excitability of DGCs activated in the home environment by shifting the input resistance towards lower values. Remarkably, that shunting inhibition is neither observed in non-activated DGCs nor in DGCs activated during training in virtual reality. In short, our results suggest that strong shunting inhibition and reduced excitability could constitute a distinctive neural signature of mature DGCs recruited in the context of the home environment.

## Introduction

The dentate gyrus (DG), an input region of the hippocampal formation processing information from the entorhinal cortex *via* the perforant path (Amaral et al., 2007), plays a crucial role in learning, memory, and spatial navigation (McNaughton and Morris, 1987; Baker et al., 2016). Memory storage and recall involve a sparse fraction of cells within the DG region (Tonegawa et al., 2015; GoodSmith et al., 2017; Hainmueller and Bartos, 2018). A few percent of dentate granule cells (DGCs) is active during behavior, while the large majority remains silent (Jung and McNaughton, 1993; Skaggs et al., 1996; Chawla et al., 2005; Neunuebel and Knierim, 2012; Tonegawa et al., 2015; Diamantaki et al., 2016; Pilz et al., 2016; Stefanelli et al., 2016; Hainmueller and Bartos, 2018; Jaeger et al., 2018; Rao-Ruiz et al., 2019). Numerous studies have described the role and the physiological properties of DGCs recruited during a behavioral task (Chawla et al., 2005; Piatti et al., 2011; Liu et al., 2012; Pardi et al., 2015; Stefanelli et al., 2016; Kirschen et al., 2017; Shevtsova et al., 2017; Hainmueller and Bartos, 2018; Pignatelli et al., 2018). Yet, that description is missing for the discreet population of DGCs that are recruited while mice are in their home cage, a condition commonly referred to as a baseline environment. In this study, we examined *ex vivo* the morphological and electrophysiological properties of subsets of DGCs that were activated when mice are maintained in their home cage without training; the properties of these cells were compared to the properties of non-activated neurons and recruited neurons during virtual reality training.

Immediate early genes (IEGs) have been widely used as neuronal activity markers in subsets of neurons that undergo plastic changes associated with learning and memory (Barth et al., 2004; Chawla et al., 2005; Stone et al., 2011; Liu et al., 2012; Czajkowski et al., 2014; Jaeger et al., 2018). Among those genes, c-fos, which has a short half-life of protein, is particularly useful for identifying neuronal ensembles with a history of recent activity (Barth, 2007). In the present study, we used a cellular tagging approach based on a transgenic mouse model in which the synthesis of the enhanced green fluorescent protein (EGFP) is controlled by the promoter of the gene *c-fos*. Accordingly, recruited neurons transiently express the EGFP, enabling their identification within a few hours for *ex vivo* electrophysiological analysis using acute brain slices (Barth et al., 2004; Czajkowski et al., 2014). Our data revealed that, when mice were maintained in their home cage (HC) without training, recruited DGCs, which were mature neurons with an extended dendritic arbor, displayed a remarkable hypoexcitability compared to non-activated cells. A higher GABA_A_ receptor-mediated shunting inhibition and a longer dendritic tree, but not Ih channel activity, contribute to the lower excitability of DGCs activated in the home environment, by shifting the input resistance towards lower values. By contrast, we did not observe a significant GABAergic shunting inhibition in non-activated DGCs and DGCs activated during training in virtual reality. We propose that shunting inhibition may be an important determinant in regulating the excitability of mature DGCs recruited in the context of the home environment.

## Materials and Methods

### Ethics

All experiments conducted on mice were performed following the European community council directives (2010/63/UE) and received approval from the French Ministry for Research, after ethical evaluation by the institutional animal care and use committee of Aix-Marseille University (protocol number: #9896-201605301121497v11).

### Mice

Adult males (*n* = 117; 97.45 ± 2.49 days old; 25.47 ± 0.26 g weight) fosGFP heterozygous mice [B6.Cg-Tg (fos/EGFP) 1–3Brth/J, Jackson Laboratory, Bar Harbor, ME, USA; RRID: IMSR_JAX:014135] were used for experiments. These mice were generated by fusing the *c-fos* promoter and the *c-fos* coding region, including exons and introns, to the coding region for EGFP, creating a fosGFP C-terminus fusion protein (Barth et al., 2004). After surgery (see below) all mice were housed in standard conditions (12 h light/dark cycles at 22–24°C, light off at 7:30 AM, housed one per cage, and food *ad libitum*) and water restricted (1 ml a day). Mice were handled before recording sessions to limit stress and experiments were performed during the dark cycle.

### Surgical Procedures

The same surgical procedure was performed on each fosGFP mouse (HC or VR). Before the surgery, mice were anesthetized with xylazine (13 mg/kg)/ketamine (66 mg/kg) in 0.9% saline and placed into a stereotactic frame. The skull was exposed and cleaned. Two screws were driven through small holes drilled in the bones and a head-bar was glued to the skull and fixed with bone cement (Heraeus Kulzer GmbH, Hanau, Germany). After 2–3 days of recovery animals were habituated to handling (1–2 days) and were water restricted (1 ml a day; Supplementary Figure 1A). If mice weight dropped below 80% of pre-water restriction weight, they were discarded from the study and placed in a cage with *ad libitum* access to food and water. Otherwise, mice were either maintained in their home cage for 2 weeks, or they underwent VR training (Supplementary Figure 1A, see below for the detailed VR training procedure). The VR trained mice and the mice maintained in their home cages were sacrificed in the morning at around 10 AM; the VR-trained mice being sacrificed 45 min after the last session (Supplementary Figure 1A). Tissue fixation and preparation of hippocampal sections or acute brain slices were carried out immediately after sacrifice for further histological and electrophysiological analyses (see below).

### Virtual Reality Set-up

Mice were trained along a linear track using virtual reality (VR) system, which is commonly used to decipher the mechanisms underlying learning and memory in the DG (Harvey et al., 2009; Dombeck et al., 2010; Ravassard et al., 2013; Schmidt-Hieber and Häusser, 2013; Hainmueller and Bartos, 2018). As that VR training procedure is performed under highly controlled conditions, trained mice acquire very stereotypical behavior (i.e., the behavior of a given VR trained mouse is similar to the behavior of another VR trained mouse, as shown Supplementary Figure 1D). Therefore, the intra-group variability is limited once mice learned the task. Mice were first habituated to be head-restrained on an air-flow-supported styrofoam ball. Then, they were trained for 18–20 sessions (30 min per session, two sessions per day; Phenosys GmbH; Berlin, Germany; Supplementary Figures 1A,B). Animals were trained to run in a 150 cm-long linear track with visual cues on the side-walls (black dots, white dots, vertical green stripes, and vertical black stripes) and distal visual cues on both sides. Those cues, which are provided through six TFT monitors surrounding the animal (JetBall-TFT, Phenosys GmbH), enabled the animals to identify their position throughout the linear track. Although mice were allowed to move the JetBall in all directions, only the movements along the longitudinal axis of the maze were recorded by the system. Upon arrival at the end of the maze, the ball was immobilized by brakes and mice received a small water drop (approximately 4 μl) through a delivery tube; consecutive rewards at the same end were not available. After having obtained the reward, mice were then able to go back by turning the jet-ball to reach the next track end (Supplementary Figure 1C). When the animals performed a sufficient number of sessions (18–20), they were anesthetized with xylazine (13 mg/kg)/ketamine (66 mg/kg) and sacrificed shortly after the training (45 min: VR condition). Calculation of mouse speed along the linear track and quantification of reward number were performed *a posteriori* using custom-developed software written in MATLAB (MathWorks, Natick, MA, USA). In the first sessions (up to seven), the mice moved slowly and erratically along the track as they learned the task (Supplementary Figure 1D). By 8–10 sessions, the mice received rewards at increasing rates over time (1.14 ± 0.05 rewards/min, *n* = 8 mice), they ran reliably back and forth along the track, and they slowed down before reaching the track end consistent with the learning of the task (Supplementary Figure 1D). We carefully monitored the VR-trained mice’s health status and wellbeing on daily basis, and we never observed any sign of chronic stress in those animals (e.g., aggressiveness, lack of grooming).

### Quantification of fosGFP^+^ Cells

The examination of cells expressing fosGFP (fosGFP^+^) was performed in hippocampal slices from animals maintained in their home cage (HC), or from animals that completed the last VR training session. Mice were deeply anesthetized with xylazine (13 mg/kg)/ketamine (66 mg/kg) prior to decapitation. The brain was then rapidly removed, and hippocampi were dissected and fixed overnight using Antigenfix (Diapath, Martinengo, Italy; home cage: *n* = 6 mice, trained in VR: *n* = 5 mice). Transverse 80 μm thick slices were cut using a Leica VT1000S vibratome (Leica Microsystems, Wetzlar, Germany). Slices were then permeabilized in blocking solution containing 5% normal goat serum (NGS, Sigma–Aldrich, Merck KGaA, Darmstadt, Germany) in 0.5% Triton for 1 h at room temperature. Slices were then incubated with the polyclonal rabbit anti-EGFP antibody (Thermo Fisher Scientific, Hampton, NH, USA) at 1:1,000 in 5% NGS in 0.5% Triton X-100 overnight at 4°C. Slices were then incubated for 2 h with the Alexa Fluor 488 secondary antibody (Invitrogen, Carlsbad, CA, USA, 1:500) and counterstained with NeuroTrace fluorescent Nissl (Invitrogen; RRID: AB_2620170) at 1:250 then coverslipped in fluoro-gel (Electron Microscopy Sciences, Hatfield, PA, USA). Fluorescent images were acquired using a confocal microscope (TCS SP5X, Leica Microsystems). Fluorescence was visualized and images were acquired with a TCS SP5X confocal microscope (Leica Microsystems, Wetzlar, Germany) equipped with a 10×/0.3 (dry) objective; thickness of the optical slice was 1 μm; Alexa Fluor 488 was imaged using an excitation wavelength of 488 nm.

Image analysis was assessed using ImageJ (NIH). The density of fosGFP^+^ cells was quantified (in cells per mm^3^) by calculating the DG area (van Praag et al., 1999) and counting the number of fosGFP^+^ cells in each slice. The percentage of fosGFP^+^ DGCs was estimated by reporting the volumetric density of fosGFP^+^ neurons (in cells per mm^3^) to the volumetric density of Prox1-positive neurons (we estimated it at 1,141,584 cells/mm^3^); the average volumetric density of Prox-1 positive cells was estimated by counting their number in a given volume (90 μm^3^, *n* = 3 mice) within the granular cell layer, and was consistent with previous observations (Amrein et al., 2004).

**Figure 1:**
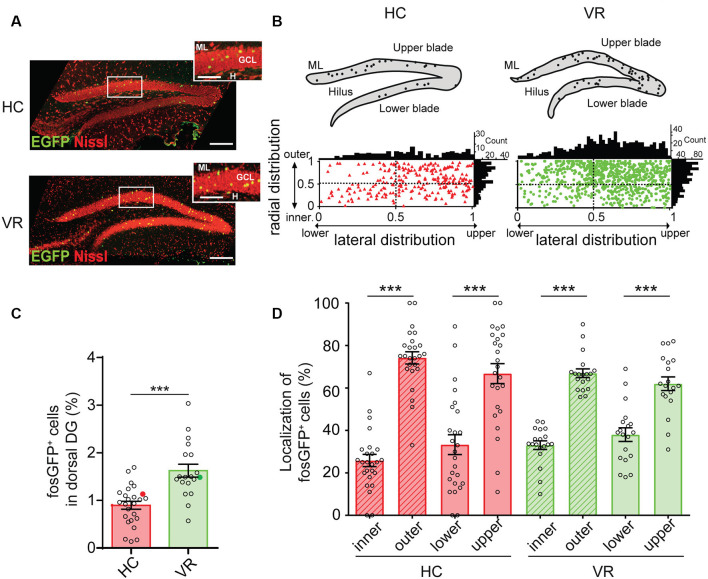
Quantification of fosGFP^+^ dentate granule cells (DGCs) and their distribution in the dorsal dentate gyrus (DG) in home cage and training conditions. **(A)** Nissl staining (red) of dorsal DG from home cage mice (HC, top) and mice trained with virtual reality (VR, bottom) using an immunostaining anti-enhanced green fluorescent protein (EGFP) antibody (green); molecular layer (ML); granule cell layer (GCL); hilus (H); scale bar 100 μm; insets show parts of DG at larger magnification; scale bar 50 μm; *z*-stack images = 26. **(B)** Bar graph displaying the percentage of fosGFP^+^ cells in dorsal DG of mice in HC and trained in VR conditions (full colored circles represent values for examples given in panel **A**). **(C)** Top: schematic illustrations of DG from confocal images of panel **(A)** with fosGFP^+^ cells represented as solid circles. Bottom: plot diagram representing the lateral and radial distributions of fosGFP^+^ cells through the dorsal DG cell layer of mice in HC (triangles) and mice trained in VR (circles); this cell layer extends laterally from the upper (suprapyramidal) blade to the lower (infrapyramidal) blade and from the outer layer (near the molecular layer) to the inner layer (near the hilus) in a radial direction. The tip of the lower blade of the GCL is defined as 0 and that of the upper blade as 1 in the lateral direction. The border between the GCL and the hilus is defined as 0 and that between the GCL and molecular layer as 1 in the radial direction. The histograms above and on the right of the square diagram indicate the number of fosGFP^+^ cells in the lateral and radial directions, respectively. **(D)** Bar graph of the radial (inner vs. outer) and lateral (lower vs. upper) distributions of fosGFP^+^ cells (%) in HC and VR conditions. In this and the following figures, data are shown as mean ± SEM. ***p < 0.0001, the statistics are shown in the Supplementary Tables 1–21.

The spatial distribution of fosGFP^+^ neurons was examined through the DG cell layer. This neuronal layer extends laterally from the upper (suprapyramidal) blade to the lower (infrapyramidal) blade and from the outer layer (near the molecular layer) to the inner layer (near the hilus) in the radial direction (Altman and Bayer, 1990; Muramatsu et al., 2007). In the lateral axis, we defined the tip of the DG lower blade as 0 and the tip of the DG upper blade as 1. Similarly, we defined in the radial direction the border between the granule cell layer (GCL) and the hilus as 0 and the border between the GCL and molecular layer as 1. Therefore, each fosGFP^+^ DGC was assigned two values between 0 and 1 corresponding to its respective position along the radial and lateral axis. The distribution of fosGFP^+^ was then plotted (Figure 1C).

The quantification of fosGFP^+^ cells was performed on the dorsal hippocampus, as this region is tightly associated with learning and memory (Fanselow and Dong, 2010). We confirmed that expression of the fosGFP transgene correlated with the synthesis of the endogenous c-fos protein using a c-fos specific antibody (Synaptic Systems GmbH, Göttingen, Germany; RRID: AB-2106755, see below); we found that virtually all EGFP-expressing neurons (i.e., fosGFP^+^ cells) were co-labeled with c-fos antibodies (100%, *n* = 18 cells, *n* = 2 mice in home cage condition; 89.7 ± 2.52%, *n* = 146 cells, *n* = 3 VR-trained mice; Supplementary Figure 2A). Immunohistochemistry performed using Prox1 antibody established that fosGFP^+^ cells corresponded to DGCs, since nearly all EGFP-positive neurons were co-labeled with Prox1 antibody (97.2 ± 2.78%, *n* = 36 cells, *n* = 3 mice in home cage condition; 98.5 ± 1.08%, *n* = 130 cells, *n* = 5 mice trained with VR; Supplementary Figure 2B).

### Immunohistochemistry

For EGFP, cfos, and Prox1 staining, 60 μm-thick slices were fixed then permeabilized in blocking solution containing 5% NGS (Sigma–Aldrich) and 0.5% Triton for 1 h at room temperature. Slices were incubated with polyclonal chicken anti-EGFP (Abcam, Cambridge, UK; RRID: AB_300798) at 1:1,000 and either with polyclonal Guinea pig anti-c-fos antibody (Synaptic Systems; RRID: AB_2106755) at 1:500 or polyclonal rabbit anti-Prox1 antibody (Millipore; Merck KGaA, Darmstadt, Germany, RRID: AB_177485) at 1:2,000 in 5% NGS in 0.5% Triton overnight at 4°C. Slices were then incubated for 2 h with Alexa Fluor 488 anti-chicken or Alexa Fluor 555 anti-guinea pig or anti-rabbit secondary antibodies (Invitrogen 1:500) and then coverslipped in fluoro-gel.

For DCX, calretinin, and calbindin staining, 60 μm-thick slices were fixed then permeabilized in blocking solution containing 5% horse serum (HS; Sigma–Aldrich) and 0.5% Triton for 2 h at room temperature. The slices were incubated with the polyclonal chicken anti-EGFP antibody (Abcam, Cambridge, UK; RRID: AB_300798) at 1:1,000 and either with polyclonal mouse anti-Prox1 antibody (Synaptic Systems; RRID: AB_2106755) at 1:500 and polyclonal rabbit anti-DCX antibody (Abcam; RRID: AB_2088478) at 1:1,000 or goat anti-Calretinin (Swant, Switzerland, RRID: CG1) at 1:1,000 or rabbit anti-Calbindin D-28k antibody (Swant, Switzerland, RRID: CB-38) at 1:5,000 in 5% HS in 0.5% Triton overnight at 4°C. Slices were then incubated for 2 h with Alexa Fluor 488 anti-chicken, Alexa Fluor 555 anti-mouse, and Alexa Fluor 647 anti-rabbit or anti-goat secondary antibodies (Invitrogen 1:500) and then coverslipped in fluoro-gel. Images of fluorescence were acquired using a TCS SP5 X confocal microscope (Leica Microsystems, Wetzlar, Germany) equipped with a 10×/0.3 (dry) and a plan-apochromat 40×/1.3 (oil immersion) objectives; thickness of the optical slice was 1 μm; Alexa Fluor 488, 555, and 647 were imaged using excitation wavelengths of 488, 555, 647 nm, respectively.

### Acute Slice Preparation

Hippocampal slices were prepared from fosGFP mice maintained in their home cage or trained with VR. Slices from mice trained with VR were cut 45 min after the last session (18–20 sessions). Animals were deeply anesthetized with xylazine (13 mg/kg)/ketamine (66 mg/kg) prior to decapitation. The brain was then rapidly removed, and transverse 350 μm thick slices were cut using a Leica VT1200S vibratome in ice-cold oxygenated (95% O_2_ and 5% CO_2_) modified artificial cerebrospinal fluid (ACSF) containing the following (in mM): 132 choline, 2.5 KCl, 1.25 NaH_2_PO_4_, 25 NaHCO_3_, 7 MgCl_2_, 0.5 CaCl_2_, and 8 D-glucose. Slices were transferred to rest at room temperature in oxygenated (95% O_2_ and 5% CO_2_) solution of ACSF containing the following (in mM): 126 NaCl, 3.5 KCl, 1.2 NaH_2_PO_4_, 26 NaHCO_3_, 1.3 MgCl_2_, 2.0 CaCl_2_, and 10 D-glucose, pH 7.4.

### Electrophysiological Recordings

Slices were placed in a submerged chamber and perfused with oxygenated ASCF (30–32°C) at a flow rate of 2–3 ml/min. Neurons were visually identified using infrared differential interference contrast microscopy (SliceScope Pro 3000M, Scientifica, Uckfield, UK). Whole-cell recordings of DGCs were obtained using the patch-clamp technique.

For current-clamp experiments, glass electrodes (resistance 6–8 MΩ) were filled with an internal solution containing the following (in mM): 130 KMeSO_4_, 5 KCl, 10 4-(2-hydroxyethyl)-1-piperazi-methanesulfonic acid, 2.5 MgATP, 0.3 NaGTP, 0.2 ethylene-glycol-tetra-acetic acid, 10 phosphocreatine, and 0.3–0.5% biocytin, pH 7.25; in this experimental condition, the equilibrium potential of chloride ions (*E*_Cl_) is around −80 mV.

For voltage-clamp experiments, glass electrodes (resistance 6–8 MΩ) were filled with an internal solution containing the following (in mM): 140 CsCl, 1 MgCl_2_, 10 HEPES, 4 NaCl, 2 Mg-ATP, 0.3 Na-GTP, 0.1 EGTA. Access resistance ranged between 15 and 30 MΩ, and the results were discarded if the access resistance changed by >20%; in this experimental condition, the equilibrium potential of chloride ions (*E*_Cl_) is around 0 mV.

Whole-cell recordings were performed in current-clamp mode using a Multiclamp 700B amplifier (Molecular Devices, Sunnyvale, CA, USA). Data were filtered at 2 kHz, digitized (20 kHz) with a Digidata 1440A (Molecular Devices) to a personal computer, and acquired using Clampex 10.1 software (PClamp, Molecular Devices). Because fosGFP transgenic animals carry multiple copies of the fosGFP transgene, the fluorescence signal is boosted, and all fosGFP^+^ cells were bright enough to be visualized even at low magnification in living neurons in acute slices (Barth et al., 2004). Therefore, neurons were considered to be fosGFP^+^ when they exhibited visually detectable fluorescence. The recordings alternated between targeting fosGFP^−^ and fosGFP^+^ neurons during an experiment. To avoid EGFP bleaching, the tissue was illuminated for a short period, typically around 5–10 s, to focus and record the image of the targeted neuron. In keeping with a previous study using fosGFP mice (Barth et al., 2004), we observed that after 3–4 h following the slicing the number of identifiable EGFP-fluorescent neurons strongly declined over time in slices, and were nearly undetectable after this period. This was consistent with the time course of fosGFP degradation similar to *in vivo* endogenous c-fos (Barth et al., 2004; Jaeger et al., 2018). Therefore, we restricted the time of fosGFP^−^ and fosGFP^+^ DGC recording to a period of 3–4 h after the mice were sacrificed.

In current-clamp mode, the electrophysiological parameters were measured after the whole-cell was established and resting membrane potential (RMP) was stable for at least 5 min. Electrophysiological properties were measured from neuronal responses to step current injections (500 ms duration) increasing from negative to positive values, and applied from a fixed membrane potential of −60 mV (Figure 3A). Input resistance (*R*_in_) was determined by plotting the membrane potential variations induced by hyperpolarizing 500 ms steps of current (from −60 to 0 pA); we did not found any significant differences when evaluating *R*_in_ at Vm = −70 mV or Vm = −60 mV (see Supplementary Tables 8, 9). The membrane time constant (*τ*_m_) was estimated from the exponential fit to the voltage response following the offset of hyperpolarizing current pulses (−40 pA amplitude, 500 ms duration). The membrane capacitance (Cm) was derived from the total dendritic length (TDL) of DGCs as previously described (Schmidt-Hieber et al., 2007), to overcome a potential bias due to GABAergic shunting inhibition on *R*_in_ (see Supplementary Tables 6, 7); using the mean TDL (see Supplementary Tables 20, 21), estimated Cm were 77.4, 102.2, 87.5, 108.9 pF for HC-fosGFP^−^ DGCs, HC-fosGFP^+^ DGCs, VR-fosGFP^−^ DGCs and VR-fosGFP^+^ DGCs, respectively. Firing frequency was studied by injecting 500 ms pulses of depolarizing current (I: from 20 pA up to 100 pA) into the cell and plotting the spike frequency (*f*) as a function of the current intensity (*f/I* plot, Figure 3B). For the analysis of action potential, the first spike evoked by a suprathreshold depolarizing current pulse was selected. A sag ratio, indicative of Ih channel activity, was calculated for current pulses (500 ms) in which the peak voltage response reached values around −120 mV; the sag ratio (%) was quantified as the difference between the peak voltage response and the steady-state voltage divided by the peak voltage response (Kowalski et al., 2016; Supplementary Figure 3, Supplementary Tables 10, 11).

**Figure 2:**
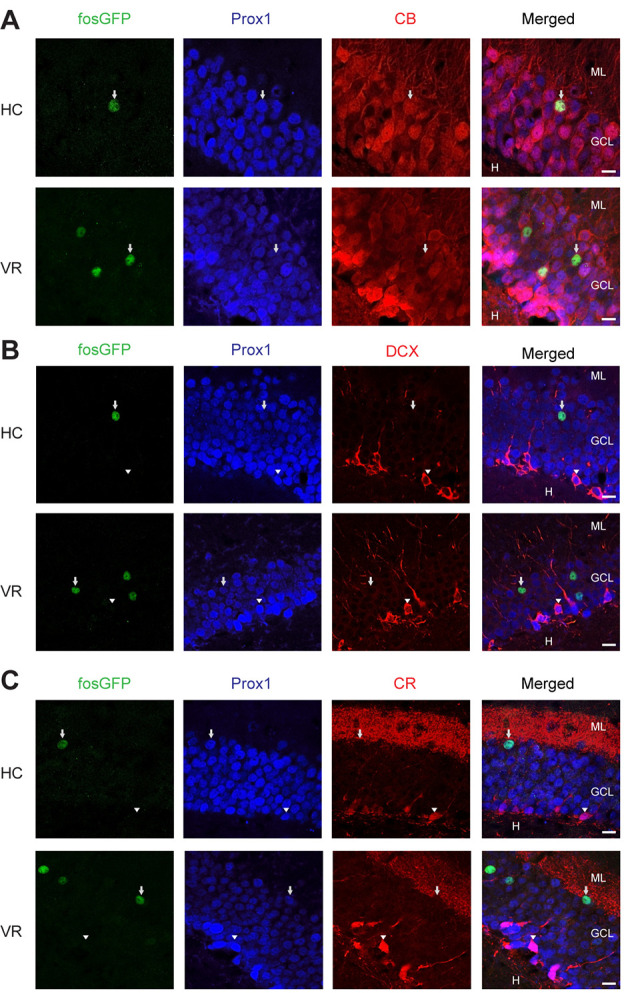
Immunoreactivity of fosGFP^+^ DGCs for calbindin, DCX, and calretinin in home cage and training conditions. Representative triple immunostaining of DGCs using anti-EGFP (first panel, arrow) antibody associated with anti-Prox1 (second panel, arrow), and anti-calbindin (CB, **A**, arrow, *z*-stack images = 13), or anti-doublecortin (DCX, **B**, arrowhead, *z-stack* images = 14), or anti-calretinin (CR, **C**, arrowhead, *z-stack* images = 9) antibodies in home cage (HC, top) and virtual reality (VR, bottom) conditions. The rightmost panels result from the merge of the other three panels; scale bar 10 μm.

**Figure 3:**
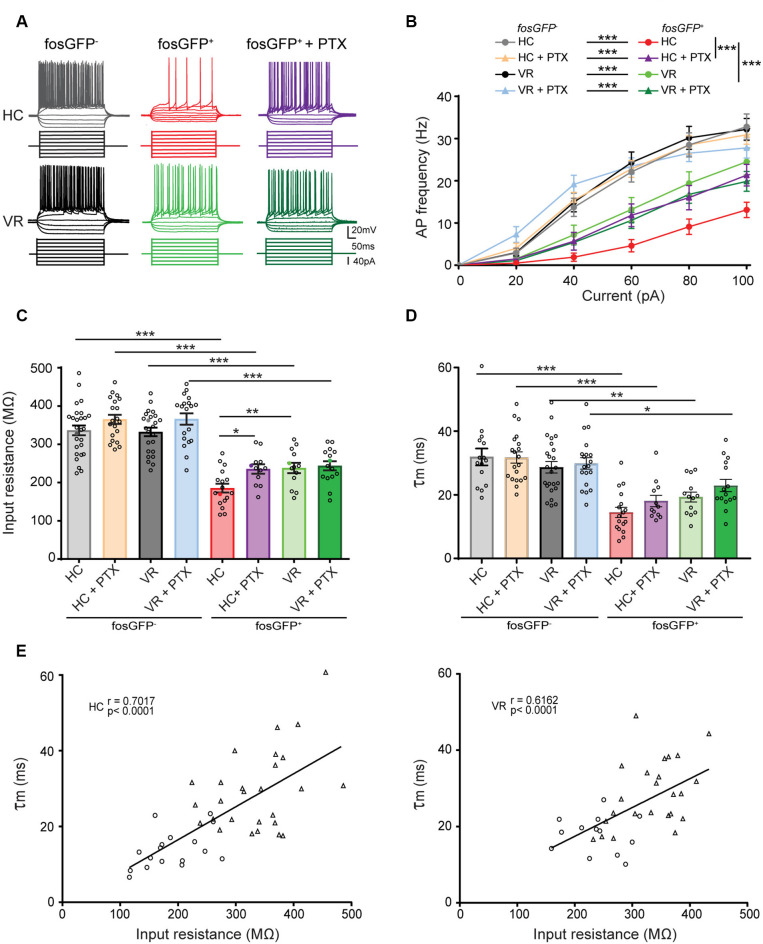
Comparative excitability of fosGFP^+^ and fosGFP^−^ DGCs in home cage and training conditions. **(A)** Representative membrane potential variations and action potential discharges of fosGFP^−^ (left), fosGFP^+^ DGCs in normal artificial cerebrospinal fluid (ACSF; middle), and fosGFP^+^ DGCs with picrotoxin (PTX, 50 μM, right) from mice in the home cage (HC, top traces) and trained mice (VR, bottom traces), elicited by 500 ms current steps varying from −60 to +100 pA by 20 pA step increments at −60 mV. **(B)** Graph showing the relationship between the mean AP frequency and the injected current for fosGFP^−^ and fosGFP^+^ DGCs in HC and VR conditions, in normal ACSF and with PTX (50–100 μM, HC + PTX, VR + PTX). **(C)** Bar graph displaying the mean input resistance of fosGFP^−^ and fosGFP^+^ DGCs in each condition. In this and following figures, each circle represents a single cell average value for bar graphs (full colored circles represent values for examples given in panel **A**). **(D)** Bar graph displaying the mean membrane time constant (*τ*_m_) of fosGFP^−^ and fosGFP^+^ DGCs in each condition. **(E)** Graphs representing the membrane time constant (*τ*_m_) as a function of the input resistance across cell populations (left panel HC, right panel VR; circles: fosGFP^+^, triangles: fosGFP^−^); line represents the significant correlation between the two parameters (HC: *r* = 0.7017, *n* = 44, *p* < 0.0001; VR: *r* = 0.6162, *n* = 37, *p* < 0.0001). *p < 0.05, **p < 0.001, ***p < 0.0001.

**Figure 4:**
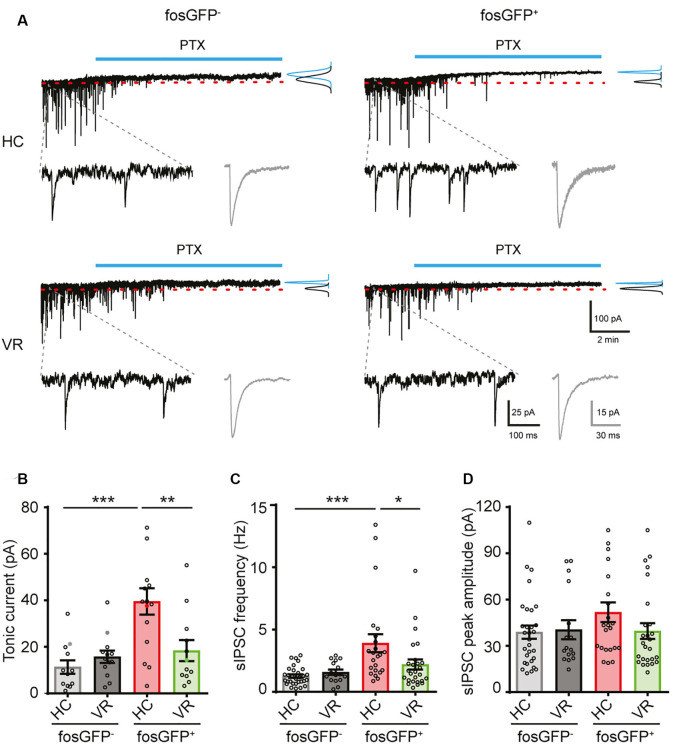
Comparative tonic and phasic GABAergic currents in fosGFP^+^ and fosGFP^−^ DGCs in home cage and training conditions. **(A)** Representative variations of membrane currents in response to PTX (50 μM, blue line) application in fosGFP^−^ (left) and fosGFP^+^ (right) DGCs from the home cage (HC, top traces) and trained mice (VR, bottom traces). Gaussian curves on the right represent the distribution of the average holding current measured over a 60 s period before (black) and after (blue) addition of PTX. Enlarged traces (bottom left) represent the spontaneous IPSCs (sIPSCs) in the different conditions; averaged sIPSCs (100 raw traces) for each condition are shown in gray (bottom right). **(B)** Bar graph representing the amplitude of tonic inhibitory current recorded in DGCs in each condition. **(C)** Bar graph representing the sIPSC frequency in DGCs in each condition. **(D)** Bar graph representing the sIPSC peak amplitude in DGCs in each condition. *p < 0.05, **p < 0.001, ***p < 0.0001.

In voltage-clamp mode, the GABA_A_ receptor-mediated current was pharmacologically isolated in the presence of AMPA, NMDA, and GABA_B_ receptor antagonists (10 μM NBQX, 40 μM D-APV, and 5 μM CGP55845, respectively). To quantify the GABA_A_ receptor-mediated tonic current, the holding current recorded during a 1 min period was analyzed by generating an all-points histogram and fitting a Gaussian distribution of this histogram (Nusser and Mody, 2002; Figure 4). Then, the median of the fitted Gaussians was used to calculate the holding currents before and during bath application of picrotoxin (PTX, GABA_A_ receptor antagonist), and to estimate the amplitude of the tonic current (Semyanov et al., 2004). PTX, Gabazine (SR-95531), NBQX, and D-APV were purchased from Tocris Bioscience (Bristol, UK). CGP55845 was purchased from HelloBio (Bristol, UK).

### Morphometric Reconstruction of Recorded Neurons

FosGFP^−^ and fosGFP^+^ neurons were labeled after intracellular diffusion of 0.3–0.5% biocytin added to the patch-clamp pipette solution (see below). At the end of the experiments, hippocampal slices were postfixed overnight at room temperature using Antigenfix. The morphology of stained neurons was revealed using 1:500 cy3-conjugated streptavidin incubated in 2% NGS in 0.5% Triton overnight at room temperature. Biocytin fluorescence was visualized and images were acquired with a TCS SP5 X confocal microscope (Leica Microsystems, Wetzlar, Germany) at magnification 20×. Three-dimensional DGC reconstructions were performed using Neurolucida Explorer Software (Micro Bright Field, Inc., Williston, VT, USA).

### Statistical Analysis

All values are given as mean ± SEM. Statistical analyses were performed using Graphpad Prism 7 (GraphPad Software, La Jolla, CA, USA). The neuronal analysis was performed blind to experimental groups. The normality of data distribution was assessed using the Shapiro–Wilk normality test. For comparison between two groups with normal distribution, the two-sample unpaired Student’s *t*-test was used, otherwise, we used the unpaired Mann–Whitney test. To investigate the relationship between the two parameters, Pearson’s correlation test was used. All tests were two-sided. For the comparison of multiple groups of two factors, we used a two-way ANOVA test; our experimental questions did not require the comparison of three factors within datasets, and the number of replicate measurements was not identical for each combination as we performed blind experiments. Bonferroni *post hoc* test was used when adequate. Statistics were provided in the text and Supplementary Tables. The level of significance was set at *p* < 0.05; exact *p* values are given, unless *p* < 0.0001 or *p* > 0.9999.

## Results

### A Sparse Population of Mature Neurons Is Recruited When Mice Are Maintained in the Home Cage

In the first set of experiments, we examined the fraction of DGCs that were recruited when mice were maintained in their home cage (HC), which is considered as a baseline environment. To that end, we used a strain of transgenic mice in which the synthesis of the fosGFP fusion protein is controlled by the promoter of the activity-dependent IEG *c-fos* (see “Materials and Methods” section). As for the endogenous c-Fos, the time course of fosGFP expression reached a peak in the 1–2 h following neuronal activation and then declined within a few hours (Barth et al., 2004; Yassin et al., 2010). Consequently, fosGFP mice enable the *ex vivo* characterization of neurons that have undergone a recent history of elevated activity *in vivo*.

When mice were maintained in HC condition, a discreet fraction of DGCs (referred as to HC-fosGFP^+^ cells) was recruited in the dorsal hippocampus, since they expressed fosGFP and the DGC marker Prox1 (0.897 ± 0.08% of DGCs, *n* = 25 slices, *n* = 6 mice; Figures 1A–C; Supplementary Figure 2; Supplementary Table 1). We then analyzed the fraction of DGCs activated in a distinct context from the home cage, which was training in a virtual reality (VR) environment (see Supplementary Figure 1, “Materials and Methods” section). In line with previous observations (Liu et al., 2012; Stefanelli et al., 2016; Kirschen et al., 2017; Shevtsova et al., 2017), the fraction of fosGFP^+^ DGCs was significantly higher in mice that experienced VR training than mice that remained in HC (1.624 ± 0.13% of VR-fosGFP^+^ DGCs, *n* = 19 slices, *n* = 5 mice, *p* < 0.0001; Figures 1A–C; Supplementary Table 1). Consistently with the previous observation showing that fosGFP expression change was transient (Barth et al., 2004; Yassin et al., 2010), the difference in the number of activated cells, which was observable in the 45 min following exposure to training in VR (VR-fosGFP^+^, see above), was no longer visible after 24 h (post-VR-fosGFP^+^; 0.83 ± 0.023% post-VR-fosGFP^+^ vs. 0.897 ± 0.08% HC-fosGFP^+^ cells, *n* = 8 slices vs., *n* = 8 slices; unpaired *t*-test *p* = 0.57).

Then, we examined the spatial distribution of fosGFP^+^ cells across the DG. We observed that a majority of recruited DGC cell bodies were located towards the upper blade of DG in the two experimental conditions (67 ± 4.7%, *n* = 25 slices, *n* = 6 mice in home cage condition; 62 ± 3.2%, *n* = 19 slices, *n* = 5 mice trained with VR; Supplementary Tables 2, 3). In addition, most fosGFP^+^ somata were situated towards the molecular layer (74 ± 2.9%, *n* = 25 slices, *n* = 6 mice in home cage condition; 67 ± 2%, *n* = 19 slices, *n* = 5 mice trained with VR; Figures 1A–D; Supplementary Tables 2, 3). That preferential location near the molecular layer suggested those fosGFP^+^ DGCs corresponded to mature DGCs. To test that hypothesis, we investigated the presence of various maturity markers (Kempermann et al., 2015) in fosGFP^+^. Our data indicated that most of fosGFP^+^ DGCs (identified using Prox1 immunochemistry) were immunopositive for calbindin (CB), a marker for mature DGCs (Kempermann et al., 2015; ranging from 86 to 100%, 87.4 ± 1.9%, *n* = 317 neurons, *n* = 15 slices, *n* = 3 mice in home cage condition; ranging from 85 to 90%, 90.5 ± 1.5%, *n* = 398 neurons, *n* = 12 slices, *n* = 2 mice in training condition; Figure 2A). By contrast, no fosGFP^+^ DGC was immunopositive for doublecortin (DCX), a marker for immature DGCs, neither in home cage condition (0 out of 37 neurons, *n* = 2 mice) nor in VR training condition (0 out of 99 neurons, *n* = 3 mice; Figure 2B). In keeping with that, we found very few Prox1-positive fosGFP^+^ DGCs that were immunopositive for calretinin (CR), a marker for immaturity of DGCs at the early post-mitotic phase (Kempermann et al., 2015; ranging from 0 to 3.8%, 0.51 ± 0.36%, *n* = 395 neurons, *n* = 15 slices, *n* = 3 mice in home cage condition; ranging from 0 to 6%, 1.66 ± 0.62%, *n* = 422 neurons, *n* = 12 slices, *n* = 2 mice in training condition; Figure 2C, Supplementary Figure 2C). Importantly, all the rare calretinin-positive fosGFP^+^ DGCs were located near the hilus as expected for immature neurons (Kempermann et al., 2015). Thus, most HC fosGFP^+^ DGCs, as VR fosGFP^+^ DGCs, were cells located near the molecular layer with mature biochemical characteristics.

In summary, our observations indicate that DGCs recruited in the home cage condition correspond to a sparse subpopulation of mature DGCs.

### Home-Cage-Activated DGCs Are Characterized by Their Hypoexcitable State

Could we find differences in the intrinsic properties between activated DGCs from mice maintained in the home cage and non-activated DGCs or activated DGCs from mice exposed to VR training conditions?

To address that question, we first examined the basic electrophysiological features of HC-fosGFP^+^ vs. HC-fosGFP^−^ using patch-clamp recordings in slices originating from mice maintained in their home cage without any training (see “Materials and Methods” section). All neurons were recorded in the upper blade of DG near the molecular layer. In current-clamp mode, we observed that HC-fosGFP^+^ DGCs displayed lower excitability than HC-fosGFP^−^ cells, as revealed by the *f/I* plot (see “Materials and Methods” section; *p* < 0.0001, Figures 3A,B; Supplementary Tables 4, 5). That difference in the excitability was not associated with any difference in action potential (AP) threshold, amplitude, or half-width (Supplementary Tables 6, 7). We then analyzed the passive membrane properties of HC-fosGFP^−^ (*n* = 27 cells) and HC-fosGFP^+^ DGCs (*n* = 17 cells). We observed that HC-fosGFP^+^ DGCs displayed a lower input resistance (*R*_in_; 185.36 ± 11.54 and 336.36 ± 12.74 MΩ in HC-fosGFP^+^ DGCs and HC-fosGFP^−^ DGCs, respectively, *p* < 0.0001; Figure 3C) associated with a faster *τ*_m_ (Figure 3D) and a higher rheobase (Supplementary Tables 6, 7). By contrast, there was no significant difference in the RMP between HC-fosGFP^+^ and HC-fosGFP^−^ DGCs (Supplementary Tables 6, 7). Therefore, DGCs recruited in the home cage condition exhibited a low excitability.

Then, we compared the intrinsic properties of HC-fosGFP^+^ DGCs with DGCs recruited or not after training in VR (i.e., VR-fosGFP^+^ and VR-fosGFP^−^ DGCs; see “Materials and Methods” section). We observed that VR-fosGFP^+^ cells displayed a higher excitability when compared with HC-fosGFP^+^ (*p* < 0.0001; Figures 3A,B; Supplementary Tables 4, 5). In keeping with this, we observed that these cells also displayed a higher *R*_in_ (237.93 ± 13.48 MΩ in 13 VR-fosGFP^+^ DGCs, *p* = 0.0066; Figure 3C) associated with a slower *τ*_m_ (Figure 3D) and a lower rheobase than HC-fosGFP^+^ DGCs (Supplementary Tables 6, 7). That higher *R*_in_ was associated with no difference in RMP, threshold, amplitude, and half-width of AP (Supplementary Tables 6, 7). Conversely, the excitability of VR-fosGFP^+^ DGCs was lower than VR-fosGFP^−^ DGCs (Figures 3A–C; Supplementary Tables 4, 5). Therefore, DGCs activated in VR condition displayed an “intermediate” state of excitability between DGCs activated in the home cage and non-activated DGCs. As expected, when plotting the membrane time constant (*τ*_m_) without* a priori* within the two different conditions (i.e., in the home cage and VR) across the pooled population of fosGFP positive and negative DGCs, we found a significant correlation between *τ*_m_ and *R*_in_ (Figure 3E). One can hypothesize that fast *τ*_m_ observed in HC-cfosGFP^+^ DGCs might allow a short window for temporal summation of synaptic inputs (Schmidt-Hieber et al., 2007; Kowalski et al., 2016).

It has been previously shown that the hyperpolarization-activated cation current *I_h_* may contribute to the modulation of DGC excitability (Stegen et al., 2012; Surges et al., 2012). Notably, *I_h_* can exert a shunting effect on excitable cells and contribute to the reduction of *R*_in_ (Fan et al., 2005; Stegen et al., 2012). We, therefore, examined *I_h_* channel activity by analyzing the sag ratio in fosGFP^+^ and fosGFP^−^ DGCs (see “Materials and Methods” section). In keeping with previous studies (Stabel et al., 1992; Lübke et al., 1998), HC- and VR- fosGFP^−^ DGCs displayed a little sag (Supplementary Figure 3). No significant difference in sag ratio was observed between HC and VR fosGFP^+^ DGCs and fosGFP^−^ DGCs (Supplementary Figure 3; Supplementary Tables 10, 11) suggesting that *I_h_* channel activity is not different between activated and non-activated DGCs in our experimental conditions.

In conclusion, our data show that DGCs recruited in the home cage condition exhibited a pronounced hypoexcitability compared with DGCs activated in VR condition (which displayed an “intermediate” excitability state) and non-activated DGCs. Moreover, our results indicate that excitability differences were unlikely to result from a distinct *I_h_* channel activity.

### Home-Cage-Recruited DGCs Display a Higher GABAergic Shunting Inhibition

It is well established that the GABA_A_ receptor-mediated tonic inhibition can play a major role in the modulation of neuronal excitability *via* a shunting effect (Brickley et al., 2001; Semyanov et al., 2004; Silver, 2010; Duguid et al., 2012; O’Neill and Sylantyev, 2018). We hypothesized that the lower excitability observed in DGCs activated in the home cage or even after VR training, when compared with non-activated DGCs, could result from a higher GABAergic shunting inhibition (Semyanov et al., 2004). To test that assumption, HC or VR fosGFP^−^ and fosGFP^+^ DGCs were recorded with or without picrotoxin (PTX, 50–100 μM), an open-channel blocker of GABA_A_ receptor well known to inhibit the GABA_A_ receptor-mediated tonic and phasic inhibition (Bright and Smart, 2013). Since PTX is not a washable compound, and to optimize the use of slices from HC and VR mice, we systematically recorded both fosGFP^−^ and fosGFP^+^ DGCs using either non-treated hippocampal slices or slices treated with PTX. Consequently, unpaired statistical analysis was made for the comparison of independent groups of DGCs recorded with or without PTX in HC or VR conditions (see “Materials and Methods” section; Supplementary Tables 6, 7). We observed that HC-fosGFP^+^ DGCs fired more in the presence of PTX, as revealed by the *f/I* plot (*p* < 0.0001, Figures 3A,B; Supplementary Tables 4, 5). That increase in excitability was associated with a significant increase in *R*_in_ (Figure 3C; Supplementary Tables 6, 7), and a decrease in rheobase when compared with HC-fosGFP^+^ DGCs recorded without PTX (Supplementary Tables 6, 7). However, that increased excitability in the presence of PTX was not associated with any significant difference in RMP, AP threshold, amplitude, or half-width (Supplementary Tables 6, 7). The effect of PTX was selective to HC-fosGFP^+^ DGCs as no noticeable effect was observed on *R*_in_ and rheobase of VR- fosGFP^+^ DGCs and fosGFP^−^ DGCs (Figure 3; Supplementary Tables 6, 7). An additional dataset of fosGFP^−^ and fosGFP^+^ DGCs in HC and VR conditions was analyzed to confirm whether similar results were obtained when *R*_in_ was evaluated in paired conditions, i.e., in the same neuron recorded before and after PTX application. Consistent with the above results, only HC-fosGFP^+^ DGCs displayed a significant increase in *R*_in_ in the presence of PTX (Supplementary Figure 4; Supplementary Tables 12, 13). Taken together, these data showed that the lower excitability observed in DGCs recruited in the home cage condition is partially due to a more significant GABA_A_ receptor-mediated shunting inhibition.

**Figure 5:**
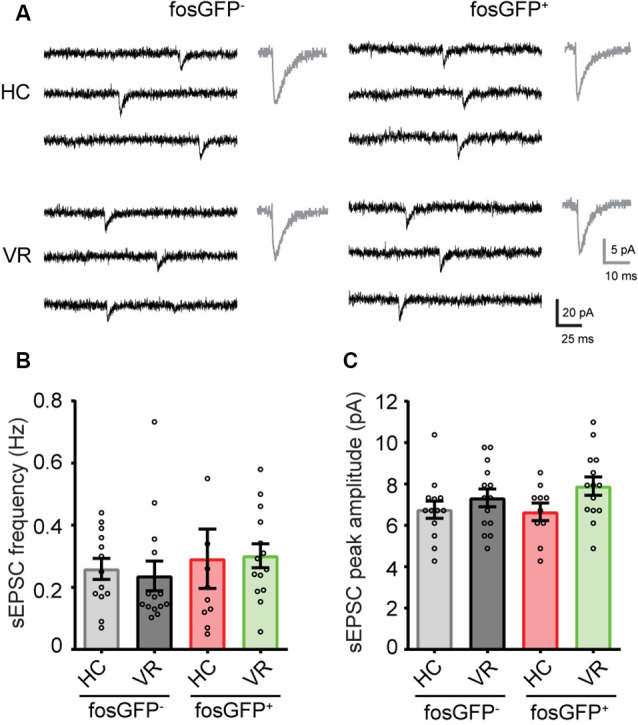
Comparative spontaneous EPSCs in fosGFP^+^ and fosGFP^−^ DGCs in home cage and training conditions. **(A)** Representative spontaneous EPSCs (sEPSCs) recorded in fosGFP^+^ and fosGFP^−^ DGCs in home cage (HC) and virtual reality (VR) conditions; averaged sEPSCs (30 raw traces) for each condition are shown in gray (right). **(B)** Bar graph representing the sEPSC frequency in DGCs in each condition. **(C)** Bar graph representing the sEPSC peak amplitude in DGCs in each condition.

We hypothesized that the differential effect of PTX could be explained by a divergence in GABA_A_-receptor tonic current amplitude between DGCs activated in the home cage, DGCs activated in the VR context, as well as non-activated DGCs. To test that hypothesis, recordings were performed in voltage-clamp mode at a holding potential of −70 mV (see “Materials and Methods” section); the GABA_A_ receptor-mediated current was pharmacologically isolated in the presence of AMPA and NMDA (10 μM NBQX and 40 μM D-APV, respectively) and GABA_B_ (5 μM CGP 55845) receptor antagonists. The amplitude of the tonic current was measured using a previously described method (Nusser and Mody, 2002; see “Materials and Methods” section). We observed a larger tonic current in HC-fosGFP^+^ DGCs (39.52 ± 5.69 pA; *n* = 15 cells) compared with HC-fosGFP^−^ DGCs (11.32 ± 2.87 pA; *n* = 12 cells; *p* < 0.0001) and VR-fosGFP^+^ DGCs (18.39 ± 4.54 pA; *n* = 12 cells; *p* = 0.002; Figures 4A,B; Supplementary Tables 14, 15). The tonic current was similar between VR-fosGFP^+^ DGCs and VR-fosGFP^−^ DGCs (Figures 4A,B; Supplementary Tables 14, 15). When the tonic GABAergic current was normalized to Cm (see “Materials and Methods” section), we also found a larger tonic current in HC-fosGFP^+^ DGCs compared with HC-fosGFP^−^ DGCs, VR-fosGFP^+^ DGCs, and VR-fosGFP^−^ DGCs (Supplementary Figure 5; Supplementary Tables 16, 17). We next monitored the GABA_A_ receptor-mediated phasic currents. We found that HC-fosGFP^+^ DGCs exhibited a higher frequency of spontaneous IPSCs compared with HC-fosGFP^−^ DGCs (*p* < 0.0001) and with VR-fosGFP^+^ DGCs (*p* = 0.0102; Figure 4C; Supplementary Tables 14, 15). That difference of IPSCs frequency was not associated with a difference in IPSCs amplitude or kinetics (Figure 4D; Supplementary Tables 14, 15). By contrast, we observed no significant difference in the IPSCs frequency between VR-fosGFP^+^ DGCs and VR-fosGFP^−^ DGCs (Figure 4C; Supplementary Tables 14, 15). We additionally examined spontaneous AMPA receptor-mediated EPSCs that were pharmacologically isolated in the presence of gabazine (5 μM) and CGP55845 (5 μM). We did not observe any significant difference in the frequency, amplitude, and kinetics of sEPSCs between all four DGC groups (HC or VR, fosGFP^+^ or fosGFP^−^; Figure 5; Supplementary Tables 18, 19).

In conclusion, our data show that DGCs recruited in the home cage condition are distinguished by a higher GABAergic shunting inhibition that leads to a significantly reduced excitability.

### Activated DGCs Display a Longer Dendritic Arbor Than Non-activated Ones

**Figure 6:**
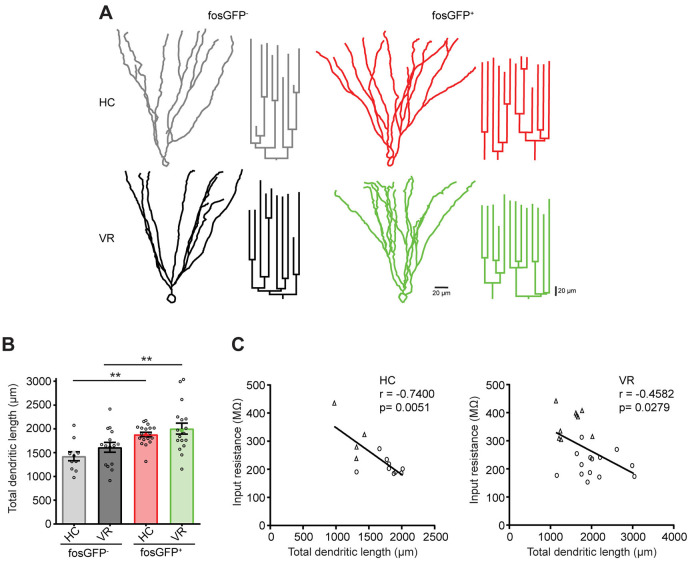
Comparative morphology of fosGFP^+^ and fosGFP^−^ DGCs in home cage and training conditions. **(A)** Reconstructions of representative fosGFP^−^ (left) and fosGFP^+^ (right) Biocytin-filled DGCs from HC (top) and VR (bottom) mice with their dendrogram (on the right). **(B)** Bar graph representing the total dendritic length (TDL; full colored circles represent values for examples given in panel **A**). **(C)** Graphs representing the input resistance of reconstructed DGCs as a function of the TDL across cell populations (left panel HC; right panel VR; circles: fosGFP^+^, triangles: fosGFP^−^); line represents the significant correlation between the two parameters (HC: *r* = −0.7400, *n* = 13, *p* = 0.0051; VR: *r* = −0.4582, *n* = 23, *p* = 0.0279). **p < 0.001.

The geometry of the dendritic arbor can participate in the modulation of the intrinsic excitability (Liu et al., 2000; Dougherty et al., 2012; Dieni et al., 2013). Therefore, we tested whether there were differences in morphologic characteristics between our four types of cells, i.e., DGCs that are activated or not (fosGFP^+^ or fosGFP^−^) in a home cage or after VR training (HC or VR). *Post hoc* morphological reconstruction of recorded neurons filled with biocytin revealed that fosGFP^+^ DGCs (recruited either in HC or in VR) displayed a more extended dendritic arbor with respect to non-activated fosGFP^−^ DGCs in the same condition (HC-fosGFP^+^: 1,881.6 ± 47.1 μm, *n* = 19 vs. HC-fosGFP^−^: 1,424.1 ± 94.4 μm, *n* = 11, *p* = 0.0038; VR-fosGFP^+^: 2,004.4 ± 116.2 μm *n* = 18 vs. VR-fosGFP^−^ 1,610.8 ± 103.6 μm, *n* = 15, *p* = 0.0072; Figures 6A,B, see “Materials and Methods” section). Conversely, HC or VR fosGFP^+^ DGCs did not display a significant difference in soma size compared with HC or VR fosGFP- DGCs (Supplementary Tables 20, 21). It has been previously reported that the TDL may be negatively correlated to *R*_in_ in some specific conditions (Liu et al., 2000; Dieni et al., 2013). In the present study, we observed a significant negative correlation between TDL and *R*_in_ without* a priori* within the two different contexts (i.e., in the home cage and VR conditions) across the pooled population of fosGFP positive and negative DGCs (Figure 6C).

In conclusion, DGCs activated in the home cage and VR conditions display an extended dendritic arbor, which correlates with a reduced *R*_in_ when compared with non-activated DGCs.

## Discussion

Previous studies have reported that a discreet fraction of DGCs is recruited in animals maintained in their home cage (Chawla et al., 2005; Liu et al., 2012; Stefanelli et al., 2016; Kirschen et al., 2017; Shevtsova et al., 2017). Here, we investigated *ex vivo* the properties of that particular ensemble of DGCs using a cfos-dependent EGFP cellular tagging approach (Barth et al., 2004). Our main finding is that DGCs activated in HC conditions, which share some common features with VR-recruited DGCs (both are mature cells with extended dendritic arbor compared to non-activated DGCs), are also discriminated by marked differences of electrophysiological properties. Especially, DGCs recruited in the home environment, which display a longer dendritic arborization, are significantly less excitable due to a lower *R*_in_ that partly results from a higher shunting inhibition. We speculate that this very low level of excitability could contribute to the sparse recruitment of mature DGCs in the home context (only ~1% DGCs activated in that condition).

It is well established that the expression of IEGs such as *c-fos* is selectively upregulated in subsets of neurons in specific brain regions associated with memory formation (Stone et al., 2011; Gore et al., 2015; Minatohara et al., 2015; Tonegawa et al., 2015). Therefore, IEGs have been largely used to tag neurons involved in memory functions and behavioral tasks. That enables the targeting of engram neurons (Ryan et al., 2015; Tonegawa et al., 2015) to describe their functional features (Barth and Poulet, 2012; Pignatelli et al., 2018). Using fosGFP mice, we identified *ex vivo* a sparse population of DGCs recruited in the dorsal hippocampus when mice were maintained in their home cage. In line with previous observations (Stone et al., 2011; Liu et al., 2012; Stefanelli et al., 2016; Kirschen et al., 2017; Shevtsova et al., 2017), we noticed a higher number of fosGFP^+^ DGCs when mice were trained in VR condition. c-Fos is a member of the AP-1 (activator protein-1) family of transcription factors and binds to DNA (Curran and Franza, 1988). Many studies reported that c-Fos expression is correlated with the establishment of engram neurons (Stone et al., 2011; Gore et al., 2015; Minatohara et al., 2015), and a recent study demonstrated the role of c-Fos in experience-dependent plasticity and learning (de Hoz et al., 2018). Therefore, the upstream signaling pathway and downstream gene targets of c-fos are of considerable interest. How the expression of these downstream genes could be translated into changes in neuronal excitability, synaptic efficacy, learning and memory remains to be established.

Since the discovery of the DG’s ability to generate new neurons throughout life (Aimone et al., 2011), it has been proposed that DG neurogenesis provides a substrate for spatial memory and pattern separation (McNaughton and Morris, 1987; Leutgeb et al., 2007; Clelland et al., 2009; Aimone et al., 2011; Sahay et al., 2011; Nakashiba et al., 2012; Neunuebel and Knierim, 2014; Kropff et al., 2015). On the other hand, it has been shown that mature DGCs play a major role in pattern completion (Nakashiba et al., 2012) and are required for the recall of familiar contexts (Vukovic et al., 2013). Along this line, a recent study demonstrates that DG place cell activity is stable over days. That observation supports the notion that mature DGCs mediate generalization between similar contexts rather than pattern separation (Hainmueller and Bartos, 2018). In the present manuscript, we first questioned the level of maturity of DGCs activated in two different contexts: home cage and VR conditions. Our data clearly showed that activated DGCs display several characteristic features of mature neurons: (i) activated DGCs were preferentially located near the molecular layer; (ii) activated DGCs were rarely co-labeled with calretinin and none were co-labeled with DCX, two different markers for immature DGCs (Kempermann et al., 2015); (iii) by contrast, we observed that virtually all activated cells were immunoreactive for calbindin, a marker for mature DGCs (Kempermann et al., 2015); (iv) activated DGCs displayed a large dendritic arbor and (v) the low mean *R*_in_ value of activated DGCs is characteristic of mature DGCs (Overstreet-Wadiche and Westbrook, 2006; Dieni et al., 2016; Save et al., 2019).

We then investigated whether the subsets of DGCs activated in the home cage condition could exhibit distinct electrophysiological properties compared to non-activated neurons and DGCs activated in VR training conditions. Our data revealed that DGCs activated in the home cage condition displayed lower excitability compared to non-activated DGCs and DGCs activated in VR training conditions. That hypoexcitability can be explained by a low *R*_in_ value, but it was associated with neither RMP nor spike threshold difference. In line with the lower *R*_in_ observed in activated DGCs in the home cage condition, we found a higher rheobase in these neurons. Nevertheless, the absolute rheobase values obtained under our experimental conditions were comparatively lower than those previously described (Save et al., 2019), since the measurements were performed at Vm = −60 mV and not at RMP. Interestingly, our data revealed that DGCs activated in VR condition were more excitable than neurons activated in the home cage condition, though those cells remain hypoexcitable concerning non-activated neurons. This is in line with previous observations showing that an increase in intrinsic excitability plays a central role in neuronal plasticity and learning processes (Daoudal and Debanne, 2003; Zhang and Linden, 2003; Barth, 2007; Epsztein et al., 2011; Sehgal et al., 2013; Yiu et al., 2014; Titley et al., 2017; Pignatelli et al., 2018; Debanne et al., 2019).

GABA_A_ receptor-mediated synaptic inhibition is a key component of sparse DGC activation (Coulter and Carlson, 2007; Dieni et al., 2013). Besides synaptic inhibition, several studies have shown that GABA_A_ receptor-mediated tonic inhibition can also play a major role in the modulation of neuronal excitability *via* a shunting effect (Brickley et al., 2001; Semyanov et al., 2004; Coulter and Carlson, 2007; Silver, 2010; Duguid et al., 2012; O’Neill and Sylantyev, 2018). Our data suggest that GABAergic shunting inhibition particularly contributes to the hypoexcitability of DGCs recruited in the home cage condition. Indeed: (i) the blockade of GABA_A_ receptors shifted *R*_in_ towards higher values and canceled the excitability difference between DGCs activated in the home cage and DGCs activated by VR training. (ii) DGCs activated in the home cage condition displayed a GABA_A_ receptor-mediated tonic current that is about four times larger than those recorded in non-activated DGCs and DGCs activated in training conditions. Furthermore, the examination of GABAergic phasic current shows that, in addition to a larger tonic current, DGCs activated in the home cage condition displayed a higher frequency of sIPSCs without a significant difference in the sEPSCs frequency. It has been recently published that spontaneously opening GABA_A_ receptors drive a tonic current shaping the kinetics of phasic inhibitory responses (O’Neill and Sylantyev, 2018). Therefore, it can be postulated that the enhanced phasic inhibition could participate in the higher tonic inhibition observed in DGCs activated in the home cage condition. It has been previously shown that δ, α4, α5, and β2 subunit-containing GABA_A_ receptors contribute to the tonic current in DGCs (Nusser and Mody, 2002; Stell and Mody, 2002; Wei et al., 2003; Chandra et al., 2006; Glykys et al., 2008; Herd et al., 2008). Further experiments should be conducted to elucidate which type of subunit-containing GABA_A_ receptors contribute to the enhanced tonic and phasic currents recorded in DGCs recruited in the home cage condition. Interestingly, in current-clamp recordings, blockade of GABA_A_ receptor-mediated tonic and phasic inhibition did not significantly change RMP in contrast to *R*_in_ in DGCs in the home cage condition. This is because the chloride reversal potential (*E*_Cl_) is close to RMP in this experimental condition (see “Materials and Methods” section and Supplementary Tables 6, 7).

Our data revealed that DGCs recruited in home cage conditions display reduced excitability in part due to an enhanced GABA signaling without a difference in the hippocampal spontaneous glutamatergic drive. Since excitatory activity is a major determinant of cFos expression by increased concentration of cytoplasmic calcium (Kim et al., 2018), this raises the question of which excitatory inputs drive the recruitment of HC-cfosGFP DGCs. It is well established that DGCs primarily receive inputs from the entorhinal cortex (Amaral et al., 2007). As experiments were performed *ex vivo* using hippocampal slices, an important part of the excitatory drive coming from the entorhinal cortex was likely absent from our recordings. Therefore, we cannot rule out the possibility that some synaptic inputs from the entorhinal cortex could provide sufficient excitatory strength *in vivo* to recruit DGCs in the home condition. On the other hand, it is well known that DGCs behave as coincidence detectors (Schmidt-Hieber et al., 2007). Therefore, another possibility is that these hypoexcitable neurons are recruited by a burst of coincident synaptic potentials that sum up in a narrow time window in the home cage condition. Previous observations suggested that neurons with low *R*_in_ displayed a fast membrane time constant, thus enabling a shorter window for temporal summation of synaptic inputs (Kowalski et al., 2016). When plotting *τ*_m_ as a function of *R*_in_ within the two different conditions (i.e., in the home cage and VR) across the pooled population of fosGFP positive and negative DGCs, we found a significant correlation between the two parameters. Accordingly, cfosGFP DGCs recruited in the home cage, which have the lowest *R*_in_, also display the fastest *τ*_m_. Thus, one can hypothesize that the fast membrane time constant observed in these HC-cfosGFP DGCs might also strengthen the coincidence detection (Schmidt-Hieber et al., 2007).

Several recent pieces of evidence suggest that DG microcircuits involving GCs and inhibitory neuron subtypes regulate GC neuron excitability and constrain their recruitment as ensembles related to a given spatial context (Stefanelli et al., 2016; Espinoza et al., 2018; Elgueta and Bartos, 2019). Recent studies revealed that the DG circuit comprised a powerful lateral inhibition (Espinoza et al., 2018) and that DGC proximal but not distal inhibition is the primary regulator of their excitability and recruitment (Elgueta and Bartos, 2019). In the present manuscript, we show that a more substantial tonic and phasic GABAergic inhibition is a distinctive feature observed in DGCs recruited in a home environment (HC-FosGFP^+^), and contribute to their pronounced hypoexcitability. Therefore, it can be hypothesized that tonic and phasic inhibition could be the resultant of DG circuit mechanisms dedicated to restraining the number of recruited neurons in the home context (Rao-Ruiz et al., 2019). Further experiments should be conducted to investigate the morpho-functional features of inhibitory neurons that drive the tonic and the phasic GABAergic inhibition received by the peculiar ensemble of DGCs activated in the home environment (HC-FosGFP^+^), and to understand better the causal role of those inhibitory neurons in DGCs sparse recruitment. Besides the participation of a GABAergic tonic current, it could be hypothesized that h-channel activity could be also involved in the modulation of *R*_in_ (Noam et al., 2011; Stegen et al., 2012; Surges et al., 2012; Shah, 2014). However, in the present study, we did not observe any significant difference in the sag ratio between non-activated and activated DGCs in the home cage and VR conditions suggesting that an h-channel activity was not involved in the shift of *R*_in_ towards lower values. Further experiments should be conducted to examine whether or not activities of other voltage-dependent and/or leak channels may participate in the modulation of *R*_in_ (Brickley et al., 2001; Marder and Goaillard, 2006).

During the development, the dendritic arbor geometry has been reported to contribute in the modulation of intrinsic properties and neuronal excitability of DGCs (Liu et al., 2000; Dieni et al., 2013). Our present study aimed at comparing the activated and the non-activated DGCs within two contexts, the home cage and training conditions, during adulthood. Our data revealed that: (i) activated mature DGCs exhibited an extended dendritic arbor and (ii) the TDL was negatively correlated with *R*_in_ (in the home cage and training conditions) compared with the non-activated DGCs. Thus, these observations suggest that the dendritic morphology of mature DGCs activated in the home cage and training conditions may play a role in retaining these neurons towards a hypoexcitable state. Interestingly, our data also suggest that extended dendritic arborization can be a predictive marker of activated neurons as previously shown during spatial exploration (Diamantaki et al., 2016). The dendritic arbor of DGCs is contacted by glutamatergic afferents including the associational/commissural and perforant pathways. Therefore, it can be hypothesized that the extended dendritic length could be associated with an enhancement of glutamatergic inputs. However, we did not observe any change in the frequency and amplitude of spontaneous EPSCs contrary to spontaneous IPSCs. This is contrasting with the classical description of homeostatic plasticity where inhibition/excitation balance and network stability tend to be maintained (Turrigiano and Nelson, 2000; Li et al., 2019). This leads us to postulate that the low excitability of activated DGCs (in the home cage and VR conditions), do not seemingly involve any homeostatic or glutamatergic plasticity mechanisms. Nevertheless, further experiments should be conducted to fully elucidate this point.

In conclusion, our data indicate that, when mice are maintained in their home cage, recruited DGCs exhibit a lower *R*_in_ and hypoexcitability. That feature is at least due to a powerful GABA_A_ receptor-mediated shunting inhibition in combination with an extended dendritic arbor that shifts *R*_in_ towards lower values. We propose that these properties could constitute a neural signature of DGCs activated in the home environment. In keeping with this, we found that DGCs activated in training conditions in VR display a higher state of excitability compared with DGCs activated in the home cage condition. Remarkably, recent work shows that engram DGCs become hyperexcitable after a recall of contextual fear memory (Pignatelli et al., 2018). Therefore, it can be speculated that DGCs display a rich repertoire of intrinsic properties depending on the context of their recruitment.

## Data Availability Statement

The original contributions presented in the study are included in the article/Supplementary Material, further inquiries can be directed to the corresponding author.

## Ethics Statement

The animal study was reviewed and approved by Institutional animal care and use committee of Aix-Marseille University (protocol number: #9896-201605301121497v11).

## Author Contributions

VC, AP, CP, and EP designed the study and conceived the experiments. AP initiated the project and therefore contributed to a big extent to the preliminary electrophysiological, histological, and behavioral observations. CP and EP continued the project, performed, and analyzed most electrophysiological, histological, and behavioral experiments detailed in the present article. AV and TS participated in the histological study and electrophysiological experiments, respectively. GM developed the software used for the acquisition and the analysis of virtual reality data. FJM participated in the confocal analysis. TM participated in the statistical analysis and the interpretation of the results. VC wrote the manuscript with input from AP, CP, EP, TM, and AV. All authors contributed to the article and approved the submitted version.

## Conflict of Interest

The authors declare that the research was conducted in the absence of any commercial or financial relationships that could be construed as a potential conflict of interest.
