# Supplementary material for: Dentate Granule Cells Recruited in the Home Environment Display Distinctive Properties
Source: Front Cell Neurosci. 2021 Jan 15;14:609123. doi: 10.3389/fncel.2020.609123 (PMC7843370; doi:10.3389/fncel.2020.609123)

Frontiers in Cellular Neuroscience - Original Article

MS Pleau - Supplementary Materials

Supplementary Table 1: Percentage of fosGFP$^+$ cells - statistical comparisons

		HC	VR
fosGFP$^+$ Cells	Mean (%)	0.897	1.624
	SEM	0.08	0.13
	n slices	25	19
	Shapiro-Wilk normality test	Yes	Yes
	unpaired t test	*** p<0.0001	

Supplementary Table 2: Lateral and radial distributions fosGFP$^+$ DGCs

		inner	outer	lower	upper
HC	Mean	25.80	74.20	66.76	33.28
	SEM	2.89	2.89	4.67	4.66
	n slices	25	25	25	25
VR	Mean	33.04	66.96	62.00	38.00
	SEM	2.05	2.05	3.20	3.20
	n slices	19	19	19	19

Supplementary Table 3: Lateral and radial distributions of fosGFP$^+$ DGCs - statistical comparisons

	Two-way ANOVA Source of Variation	F (DFn, DFd)	p value	post-hoc Bonferroni p value
HC inner / HC outer	radial distribution	F (1, 84) = 239.0	p< 0.0001	*** p< 0.0001
VR inner / VR outer	radial distribution	F (1, 84) = 239.0	p< 0.0001	*** p< 0.0001
HC inner / VR inner	behavior	F (1, 84) = 1.426e-013	p> 0.9999	none
HC outer / VR outer	behavior	F (1, 84) = 1.426e-013	p> 0.9999	none
HC upper / HC lower	lateral distribution	F (1, 84) = 45.19	p< 0.0001	*** p< 0.0001
VR upper / VR lower	lateral distribution	F (1, 84) = 45.19	p< 0.0001	*** p=0.0007

HC upper / VR upper	behavior	F (1, 84) = 2.188e-005	p = 0.9963	none
HC lower / VR lower	behavior	F (1, 84) = 2.188e-005	p = 0.9963	none

Supplementary Table 4: f/I

			Injected current (pA)					
			0	20	40	60	80	100
Action Potential frequency (Hz)	HC fosGFP$^-$	Mean	0	2.889	13.7	22.15	28.52	32.8
		SEM	0	1.146	2.153	2.413	2.848	3.033
		n cells	27	27	27	27	27	27
	HC fosGFP$^-$ PTX	Mean	0	4	15.4	22.8	28.5	30.9
		SEM	0	1.196	1.836	1.976	2.166	2.273
		n cells	20	20	20	20	20	20
	VR fosGFP$^-$	Mean	0	3	14.92	24.33	30.17	32.18
		SEM	0	0.7518	2.268	2.49	2.705	2.588
		n cells	24	24	24	24	24	24
	VR fosGFP$^-$ PTX	Mean	0	7.263	19.16	23.47	26.53	27.79
		SEM	0	1.868	2.159	1.97	1.981	2.418
		n cells	19	19	19	19	19	19
	HC fosGFP$^+$	Mean	0	0.4706	1.882	4.588	9.059	13.06
		SEM	0	0.4706	0.9772	1.473	1.816	1.832
		n cells	17	17	17	17	17	17
	HC fosGFP$^+$ PTX	Mean	0	1.5	5.667	11.83	16	21.33
		SEM	0	0.8919	2.101	2.702	2.881	2.562
		n cells	12	12	12	12	12	12
	VR fosGFP$^+$	Mean	0	1.538	7.231	13.23	19.38	24.46
		SEM	0	0.8815	2.271	2.833	2.74	3.073
		n cells	13	13	13	13	13	13
	VR fosGFP+ PTX	Mean	0	0.9333	5.333	10.8	16.53	19.73
		SEM	0	0.8016	1.629	1.699	1.856	2.046
		n cells	15	15	15	15	15	15

Supplementary Table 5: f/I – statistical comparisons

	Two-way ANOVA Source of Variation	F (DFn. DFd)	p value
HC fosGFP^{-} / HC fosGFP^{-} PTX	PTX	F (1.268) = 0.04566	p=0.8310
VR fosGFP^{-} / VR fosGFP^{-} PTX	PTX	F (1.244) = 0.003046	p=0.9560
HC fosGFP^{+} / HC fosGFP^{+} PTX	PTX	F (1.162) = 22.31	*** p<0.0001
VR fosGFP^{+} / VR fosGFP^{+} PTX	PTX	F (1.156) = 3.671	p=0.0572
HC fosGFP^{-} / VR fosGFP^{-}	behavior	F (1.290) = 0.3759	p=0.5403
HC fosGFP^{+} / VR fosGFP^{+}	behavior	F (1.168) = 37.01	*** p<0.0001
HC fosGFP^{-} PTX / VR fosGFP^{-} PTX	behavior	F (1.222) = 0.1693	p=0.6811
HC fosGFP^{+} PTX / VR fosGFP^{+} PTX	behavior	F (1.150) = 0.2302	p=0.6321
HC fosGFP^{-} / HC fosGFP^{+}	fosGFP	F (1.250) = 98.21	*** p<0.0001
VR fosGFP^{-} / VR fosGFP^{+}	fosGFP	F (1.208) = 23.57	*** p<0.0001
HC fosGFP^{-} PTX / HC fosGFP^{+} PTX	fosGFP	F (1.180) = 43.21	*** p<0.0001
VR fosGFP^{-} PTX / VR fosGFP^{+} PTX	fosGFP	F (1.192) = 67.03	*** p<0.0001

Supplementary Table 6: Intrinsic properties

			RMP (mV)	R input (MΩ)	τ_m (ms)	Rheobase (pA)	AP Threshold (mV)	AP amplitude (mV)	AP HW (ms)
HC	fos GFP$^-$	Mean	-78.30	336.40	31.90	34.81	-42.26	77.65	1.22
		SEM	1.10	12.74	2.65	2.29	1.14	2.02	0.04
		n cells	23	27	27	27	27	27	27
	fos GFP$^+$	Mean	-75.32	185.40	14.47	70.59	-39.43	75.56	1.25
		SEM	1.23	11.54	1.604	8.07	1.95	2.35	0.07
		n cells	13	17	17	17	17	17	17
VR	fos GFP$^-$	Mean	-79.25	332.40	28.65	30.83	-41.39	78.39	1.18
		SEM	1.35	11.29	1.81	2.40	0.93	1.52	0.03
		n cells	23	24	24	24	24	24	24
	fos GFP$^+$	Mean	-77.86	237.90	19.30	43.08	-40.19	73.66	1.12
		SEM	1.83	13.48	1.57	4.99	1.78	2.84	0.06
		n cells	11	13	13	13	13	13	13
HC + PTX	fos GFP$^-$	Mean	-78.25	365.28	31.73	30.00	-41.84	75.14	1.26
		SEM	0.93	11.97	1.77	2.29	1.42	2.20	0.04
		n cells	20	20	20	20	20	20	20
	fos GFP$^+$	Mean	-77.09	235.35	18.04	46.67	-40.24	75.24	1.24
		SEM	1.15	12.23	1.78	5.69	2.13	3.61	0.06
		n cells	12	12	12	12	12	12	12
VR + PTX	fos GFP$^-$	Mean	-77.43	366.04	28.86	26.32	-42.91	80.38	1.23
		SEM	0.76	14.81	1.81	2.19	1.25	1.84	0.04
		n cells	19	19	19	19	19	19	19
	fos GFP$^+$	Mean	-77.49	243.67	22.89	46.67	-40.68	78.93	1.25
		SEM	1.04	11.92	1.935	4.22	1.98	2.33	0.04
		n cells	15	15	15	15	15	15	15

Supplementary Table 7: Intrinsic properties - statistical comparisons

		Two-way ANOVA Source of Variation	F (DFn. DFd)	ANOVA p value	post-hoc Bonferroni p value
RMP	HC fosGFP$^-$ / HC fosGFP$^+$	fosGFP	F (1. 64) = 3.176	p=0.0795	none
	HC fosGFP$^-$ PTX / HC fosGFP$^+$ PTX	fosGFP	F (1. 64) = 3.176	p=0.0795	none
	VR fosGFP$^-$ / VR fosGFP$^+$	fosGFP	F (1. 64) = 0.2579	p=0.6133	none
	VR fosGFP$^-$ PTX / VR fosGFP$^+$ PTX	fosGFP	F (1. 64) = 0.2579	p=0.6133	none
	HC fosGFP$^+$ / VR fosGFP$^+$	behavior	F (1. 47) = 1.264	p=0.2666	none
	HC fosGFP$^+$ PTX / VR fosGFP$^+$ PTX	behavior	F (1. 47) = 1.264	p=0.2666	none
	HC fosGFP$^-$ / VR fosGFP$^-$	behavior	F (1. 81) = 0.003189	p=0.9551	none
	HC fosGFP$^-$ PTX / VR fosGFP$^-$ PTX	behavior	F (1. 81) = 0.003189	p=0.9551	none
	HC fosGFP$^+$ / HC fosGFP$^+$ PTX	PTX	F (1. 47) = 0.2841	p=0.5965	none
	VR fosGFP$^+$ / VR fosGFP$^+$ PTX	PTX	F (1. 47) = 0.2841	p=0.5965	none
	HC fosGFP$^-$ / HC fosGFP$^-$ PTX	PTX	F (1. 81) = 0.7247	p=0.3971	none
	VR fosGFP$^-$ / VR fosGFP$^-$ PTX	PTX	F (1. 81) = 0.7247	p=0.3971	none
Input resistance	HC fosGFP$^-$ / HC fosGFP$^+$	fosGFP	F (1. 72) = 110.5	p<0.0001	*** p<0.0001
	HC fosGFP$^-$ PTX / HC fosGFP$^+$ PTX	fosGFP	F (1. 72) = 110.5	p<0.0001	*** p<0.0001
	VR fosGFP$^-$ / VR fosGFP$^+$	fosGFP	F (1. 67) = 65.04	p<0.0001	*** p<0.0001
	VR fosGFP$^-$ PTX / VR fosGFP$^+$ PTX	fosGFP	F (1. 67) = 65.04	p<0.0001	*** p<0.0001

	HC fosGFP$^+$ / VR fosGFP$^+$	behavior	F (1. 53) = 6.026	p=0.0174	**p=0.0066
	HC fosGFP$^+$ PTX / VR fosGFP$^+$ PTX	behavior	F (1. 53) = 6.026	p=0.0174	p>0.9999
	HC fosGFP$^-$ / VR fosGFP$^-$	behavior	F (1. 86) = 0.01569	p=0.9006	none
	HC fosGFP$^-$ PTX / VR fosGFP$^-$ PTX	behavior	F (1. 86) = 0.01569	p=0.9006	none
	HC fosGFP$^+$ / HC fosGFP$^+$ PTX	PTX	F (1. 53) = 5.046	p=0.0289	* p=0.0122
	VR fosGFP$^+$ / VR fosGFP$^+$ PTX	PTX	F (1. 53) = 5.046	p=0.0289	p>0.9999
	HC fosGFP$^-$ / HC fosGFP$^-$ PTX	PTX	F (1. 86) = 5.921	p=0.0170	p=0.2166
	VR fosGFP$^-$ / VR fosGFP$^-$ PTX	PTX	F (1. 86) = 5.921	p=0.0170	p=0.1459
τ_m	HC fosGFP$^-$ / HC fosGFP$^+$	fosGFP	F (1, 60) = 59.54	p<0.0001	*** p<0.0001
	HC fosGFP$^-$ PTX / HC fosGFP$^+$ PTX	fosGFP	F (1, 60) = 59.54	p<0.0001	*** p<0.0001
	VR fosGFP$^-$ / VR fosGFP$^+$	fosGFP	F (1, 67) = 34.18	p<0.0001	** p=0.0046
	VR fosGFP$^-$ PTX / VR fosGFP$^+$ PTX	fosGFP	F (1. 67) = 18.27	p<0.0001	* p=0.0241
	HC fosGFP$^+$ / VR fosGFP$^+$	behavior	F (1, 53) = 7.581	p=0.0081	p=0.1029
	HC fosGFP$^+$ PTX / VR fosGFP$^+$ PTX	behavior	F (1, 53) = 7.581	p=0.0081	p=0.1246
	HC fosGFP$^-$ / VR fosGFP$^-$	behavior	F (1, 74) = 4.588	p=0.4895	none
	HC fosGFP$^-$ PTX / VR fosGFP$^-$ PTX	behavior	F (1, 74) = 1.648	p=0.2033	none
	HC fosGFP$^+$ / HC fosGFP$^+$ PTX	interaction (PTX x fosGFP)	F (1, 60) = 0.7085	p=0.4033	none
	VR fosGFP$^+$ / VR fosGFP$^+$ PTX	interaction (PTX x fosGFP)	F (1, 67) = 1.581	p=0.2130	none

	HC fosGFP$^-$ / HC fosGFP$^-$ PTX	PTX	F (1, 60) = 0.7085	p=0.4033	none
	VR fosGFP$^-$ / VR fosGFP$^-$ PTX	PTX	F (1, 67) = 1.581	p=0.2130	none
Rheobase	HC fosGFP$^-$ / HC fosGFP$^+$	fosGFP	F (1. 72) = 31.26	p<0.0001	*** p<0.0001
	HC fosGFP$^-$ PTX / HC fosGFP$^+$ PTX	fosGFP	F (1. 72) = 31.26	p<0.0001	* p=0.0453
	VR fosGFP$^-$ / VR fosGFP$^+$	fosGFP	F (1. 67) = 24.06	p<0.0001	* p=0.0223
	VR fosGFP$^-$ PTX / VR fosGFP$^+$ PTX	fosGFP	F (1. 67) = 24.06	p<0.0001	***p=0.0001
	HC fosGFP$^+$ / VR fosGFP$^+$	behavior	F (1. 53) = 4.747	p=0.0338	** p=0.0052
	HC fosGFP$^+$ PTX / VR fosGFP$^+$ PTX	behavior	F (1. 53) = 4.747	p=0.0338	p>0.9999
	HC fosGFP$^-$ / VR fosGFP$^-$	behavior	F (1. 86) = 2.654	p=0.1069	none
	HC fosGFP$^-$ PTX / VR fosGFP$^-$ PTX	behavior	F (1. 86) = 2.654	p=0.1069	none
	HC fosGFP$^+$ / HC fosGFP$^+$ PTX	interaction (PTX x fosGFP)	F (1. 53) = 4.747	p=0.0338	* p=0.0193
	VR fosGFP$^+$ / VR fosGFP$^+$ PTX	interaction (PTX x fosGFP)	F (1. 53) = 4.747	p=0.0338	p>0.9999
	HC fosGFP$^-$ / HC fosGFP$^-$ PTX	PTX	F (1. 86) = 2.654	p=0.1069	none
	VR fosGFP$^-$ / VR fosGFP$^-$ PTX	PTX	F (1. 86) = 2.654	p=0.1069	none
AP Threshold	HC fosGFP$^-$ / HC fosGFP$^+$	fosGFP	F (1. 72) = 1.855	p=0.1775	none
	HC fosGFP$^-$ PTX / HC fosGFP$^+$ PTX	fosGFP	F (1. 72) = 1.855	p=0.1775	none
	VR fosGFP$^-$ / VR fosGFP$^+$	fosGFP	F (1. 67) = 1.42	p=0.2375	none
	VR fosGFP$^-$ PTX / VR fosGFP$^+$ PTX	fosGFP	F (1. 67) = 1.42	p=0.2375	none

	HC fosGFP$^+$ / VR fosGFP$^+$	behavior	F (1. 53) = 0.09076	p=0.7644	none
	HC fosGFP$^+$ PTX / VR fosGFP$^+$ PTX	behavior	F (1. 53) = 0.09076	p=0.7644	none
	HC fosGFP$^-$ / VR fosGFP$^-$	behavior	F (1. 86) = 0.006899	p=0.9340	none
	HC fosGFP$^-$ PTX / VR fosGFP$^-$ PTX	behavior	F (1. 86) = 0.006899	p=0.9340	none
	HC fosGFP$^+$ / HC fosGFP$^+$ PTX	PTX	F (1. 53) = 0.1066	p=0.7454	none
	VR fosGFP$^+$ / VR fosGFP$^+$ PTX	PTX	F (1. 53) = 0.1066	p=0.7454	none
	HC fosGFP$^-$ / HC fosGFP$^-$ PTX	PTX	F (1. 86) = 0.2109	p=0.6472	none
	VR fosGFP$^-$ / VR fosGFP$^-$ PTX	PTX	F (1. 86) = 0.2109	p=0.6472	none
AP Amplitude	HC fosGFP$^-$ / HC fosGFP$^+$	fosGFP	F (1. 72) = 0.1554	p=0.6946	none
	HC fosGFP$^-$ PTX / HC fosGFP$^+$ PTX	fosGFP	F (1. 72) = 0.1554	p=0.6946	none
	VR fosGFP$^-$ / VR fosGFP$^+$	fosGFP	F (1. 67) = 2.218	p=0.1411	none
	VR fosGFP$^-$ PTX / VR fosGFP$^+$ PTX	fosGFP	F (1. 67) = 2.218	p=0.1411	none
	HC fosGFP$^+$ / VR fosGFP$^+$	behavior	F (1. 53) = 0.1062	p=0.7459	none
	HC fosGFP$^+$ PTX / VR fosGFP$^+$ PTX	behavior	F (1. 53) = 0.1062	p=0.7459	none
	HC fosGFP$^-$ / VR fosGFP$^-$	behavior	F (1. 86) = 2.369	p=0.1274	none
	HC fosGFP$^-$ PTX / VR fosGFP$^-$ PTX	behavior	F (1. 86) = 2.369	p=0.1274	none
	HC fosGFP$^+$ / HC fosGFP$^+$ PTX	PTX	F (1. 53) = 0.8071	p=0.3730	none
	VR fosGFP$^+$ / VR fosGFP$^+$ PTX	PTX	F (1. 53) = 0.8071	p=0.3730	none
	HC fosGFP$^-$ / HC fosGFP$^-$ PTX	PTX	F (1. 86) = 0.01779	p=0.8942	none

	VR fosGFP$^-$ / VR fosGFP$^-$ PTX	PTX	F (1. 86) = 0.01779	p=0.8942	none
AP HW	HC fosGFP$^-$ / HC fosGFP$^+$	fosGFP	F (1. 72) = 0.003354	p=0.9540	none
	HC fosGFP$^-$ PTX / HC fosGFP$^+$ PTX	fosGFP	F (1. 72) = 0.003354	p=0.9540	none
	VR fosGFP$^-$ / VR fosGFP$^+$	fosGFP	F (1. 67) = 0.2742	p=0.6023	none
	VR fosGFP$^-$ PTX / VR fosGFP$^+$ PTX	fosGFP	F (1. 67) = 0.2742	p=0.6023	none
	HC fosGFP$^+$ / VR fosGFP$^+$	behavior	F (1. 53) = 0.9522	p=0.3336	none
	HC fosGFP$^+$ PTX / VR fosGFP$^+$ PTX	behavior	F (1. 53) = 0.9522	p=0.3336	none
	HC fosGFP$^-$ / VR fosGFP$^-$	behavior	F (1. 86) = 0.8155	p=0.3690	none
	HC fosGFP$^-$ PTX / VR fosGFP$^-$ PTX	behavior	F (1. 86) = 0.8155	p=0.3690	none
	HC fosGFP$^+$ / HC fosGFP$^+$ PTX	PTX	F (1. 53) = 0.9398	p=0.3367	none
	VR fosGFP$^+$ / VR fosGFP$^+$ PTX	PTX	F (1. 53) = 0.9398	p=0.3367	none
	HC fosGFP$^-$ / HC fosGFP$^-$ PTX	PTX	F (1. 86) = 1.908	p=0.1707	none
	VR fosGFP$^-$ / VR fosGFP$^-$ PTX	PTX	F (1. 86) = 1.908	p=0.1707	none

Supplementary Table 8: R_{in} measured at Vm=-70mV and Vm=-60mV

			R input (MΩ)
HC fosGFP^{-}	Vm= -70mV	Mean	359.1
		SEM	34.12
		n cells	10
	Vm= -60mV	Mean	336.4.1
		SEM	12.74
		n cells	27
HC fosGFP^{+}	Vm= -70mV	Mean	176.3
		SEM	17.92
		n cells	7
	Vm= -60mV	Mean	185.4
		SEM	11.54
		n cells	17
VR fosGFP^{-}	Vm= -70mV	Mean	374.4
		SEM	17.34
		n cells	22
	Vm= -60mV	Mean	332.4
		SEM	11.29
		n cells	24
VR fosGFP^{+}	Vm= -70mV	Mean	244.3
		SEM	12.31
		n cells	15
	Vm= -60mV	Mean	237.9
		SEM	13.48
		n cells	13

Supplementary Table 9: R_{in} measured at Vm=-70mV and Vm=-60mV - Two-way Anova statistical comparisons

R_{in} at Vm = -70mV vs R_{in} at Vm = -60mV	Two-way ANOVA Source of Variation	F (DFn. DFd)	ANOVA p value	post-hoc Bonferroni p value
HC fosGFP$^-$	Vm	F (1, 127) = 1.599	P=0.2083	0.8181
HC fosGFP$^+$	Vm	F (1, 127) = 1.599	P=0.2083	0.9965
VR fosGFP$^-$	Vm	F (1, 127) = 1.599	P=0.2083	0.1165
VR fosGFP$^+$	Vm	F (1, 127) = 1.599	P=0.2083	0.9982

Supplementary table 10: Sag ratio

			Sag ratio (%)
HC	fosGFP$^-$	Mean	1.94
		SEM	0.22
		n cells	21
	fosGFP$^+$	Mean	1.83
		SEM	0.23
		n cells	19
VR	fosGFP$^-$	Mean	1.75
		SEM	0.21
		n cells	19
	fosGFP$^+$	Mean	1.44
		SEM	0.25
		n cells	16

Supplementary table 11: Sag ratio - statistical comparisons

	Two-way ANOVA Source of variation	F (DFn, DFd)	p value
HC fosGFP$^-$ / HC fosGFP$^+$	fosGFP	F (1, 71) = 0.862	p=0.3563
VR fosGFP$^-$ / VR fosGFP$^+$	fosGFP	F (1, 71) = 0.862	p=0.3563
HC fosGFP$^+$ / VR fosGFP$^+$	behavior	F (1, 71) = 1.579	p=0.2130
HC fosGFP$^-$ / VR fosGFP$^-$	behavior	F (1, 71) = 1.579	p=0.2130

Supplementary table 12: Paired analysis of Ri before and after PTX

			Control	PTX	Variation rate (%)
HC	fosGFP$^-$	Mean	365.9	382.0	4.26
		SEM	29.77	33.09	1.30
		n cells	5	5	5
	fosGFP$^+$	Mean	196.0	253.4	30.91
		SEM	21.05	22.56	4.01
		n cells	7	7	7
VR	fosGFP$^-$	Mean	363.2	380.3	5.81
		SEM	50.67	48.18	2.71
		n cells	6	6	6
	fosGFP$^+$	Mean	276.6	289.5	5.14
		SEM	39.67	39.76	1.41
		n cells	5	5	5

Supplementary table 13: Paired analysis of Ri before and after PTX - statistical comparisons

	Repeated measures two-way ANOVA Source of Variation	F (DFn. DFd)	ANOVA p value	post-hoc Bonferroni p value
HC fosGFP$^+$ / HC fosGFP$^+$ PTX	PTX	F (1, 19) = 56.81	p<0.0001	p= 0.1589
VR fosGFP$^+$ / VR fosGFP$^+$ PTX	PTX	F (1, 19) = 56.81	p<0.0001	*** p<0.0001
HC fosGFP$^-$ / HC fosGFP$^-$ PTX	PTX	F (1, 19) = 56.81	p<0.0001	p= 0.3716
VR fosGFP$^-$ / VR fosGFP$^-$ PTX	PTX	F (1, 19) = 56.81	p<0.0001	p= 0.0755

Supplementary Table 14: GABAergic tonic current and sIPSC

			Tonic current (pA)	sIPSC Frequency (Hz)	sIPSC Amplitude (pA)	sIPSC Rise time (ms)	sIPSC Decay time (ms)	sIPSC Rise slope (pA/ms)
HC	fosGFP$^-$	Mean	11.32	1.27	38.88	1.83	10.58	35.20
		SEM	2.87	0.14	4.27	0.23	1.33	6.60
		n cells	12	30	30	30	30	30
	fosGFP$^+$	Mean	39.52	3.90	51.81	1.65	10.70	41.47
		SEM	5.69	0.72	6.39	0.18	1.61	8.89
		n cells	15	23	23	23	23	23
VR	fosGFP$^-$	Mean	15.72	1.57	40.45	1.85	11.10	29.96
		SEM	2.63	0.21	6.20	0.25	1.91	7.26
		n cells	13	16	16	16	16	16
	fosGFP$^+$	Mean	18.39	2.19	39.59	1.81	9.93	33.14
		SEM	4.54	0.41	5.11	0.23	1.14	7.25
		n cells	12	26	26	26	26	26

Supplementary Table 15: GABAergic tonic current and sIPSC - statistical comparisons

		Two-way ANOVA Source of variation	F (DFn. DFd)	p value	post-hoc Bonferroni p value
Tonic current	HC fosGFP$^-$ / HC fosGFP$^+$	fosGFP	F (1. 48) = 12.64	p=0.0009	*** p<0.0001
	VR fosGFP$^-$ / VR fosGFP$^+$	fosGFP	F (1. 48) = 12.64	p=0.0009	p>0.9999
	HC fosGFP$^+$ / VR fosGFP$^+$	interaction (behaviour x fosGFP)	F (1. 48) = 8.642	p=0.005	** p=0.002
	HC fosGFP- / VR fosGFP$^-$	interaction (behaviour x fosGFP)	F (1. 48) = 8.642	p=0.005	p=0.969
sIPSC frequency	HC fosGFP$^-$ / HC fosGFP$^+$	fosGFP	F (1. 91) = 13.74	p=0.0004	*** p<0.0001
	VR fosGFP$^-$ / VR fosGFP$^+$	fosGFP	F (1. 91) = 13.74	p=0.0004	p=0.7036
	HC fosGFP$^+$ / VR fosGFP$^+$	interaction (behavior x fosGFP)	F (1. 91) = 5.274	p=0.0239	* p=0.0102
	HC fosGFP$^-$ / VR fosGFP$^-$	interaction (behavior x fosGFP)	F (1. 91) = 5.274	p=0.0239	p>0.9999
sIPSC amplitude	HC fosGFP$^-$ / HC fosGFP$^+$	fosGFP	F (1. 91) = 1.19	p=0.2782	none
	VR fosGFP$^-$ / VR fosGFP$^+$	fosGFP	F (1. 91) = 1.19	p=0.2782	none
	HC fosGFP$^+$ / VR fosGFP$^+$	behavior	F (1. 91) = 0.9265	p=0.3383	none
	HC fosGFP$^-$ / VR fosGFP$^-$	behavior	F (1. 91) = 0.9265	p=0.3383	none
sIPSC rise time	HC fosGFP$^-$ / HC fosGFP$^+$	fosGFP	F (1. 91) = 0.2133	p=0.6453	none
	VR fosGFP$^-$ / VR fosGFP$^+$	fosGFP	F (1. 91) = 0.2133	p=0.6453	none
	HC fosGFP$^+$ / VR fosGFP$^+$	behavior	F (1. 91) = 0.1586	p=0.6914	none

	HC fosGFP^{-} / VR fosGFP^{-}	behavior	F (1. 91) = 0.1586	p=0.6914	none
sIPSC decay time	HC fosGFP^{-} / HC fosGFP^{+}	fosGFP	F (1. 91) = 0.1252	p=0.7243	none
	VR fosGFP^{-} / VR fosGFP^{+}	fosGFP	F (1. 91) = 0.1252	p=0.7243	none
	HC fosGFP^{+} / VR fosGFP^{+}	behavior	F (1. 91) = 0.0072	p=0.9323	none
	HC fosGFP^{-} / VR fosGFP^{-}	behavior	F (1. 91) = 0.0072	p=0.9323	none
sIPSC rise slope	HC fosGFP^{-} / HC fosGFP^{+}	fosGFP	F (1. 91) = 0.1252	p=0.3664	none
	VR fosGFP^{-} / VR fosGFP^{+}	fosGFP	F (1. 91) = 0.1252	p=0.3664	none
	HC fosGFP^{+} / VR fosGFP^{+}	behavior	F (1. 91) = 0.0072	p=0.7560	none
	HC fosGFP^{-} / VR fosGFP^{-}	behavior	F (1. 91) = 0.0072	p=0.7560	none

Supplementary Table 16: GABAergic tonic (pA/pF)

HC	fosGFP^{-}	Mean	0.15
		SEM	0.037
		n cells	12
	fosGFP^{+}	Mean	0.39
		SEM	0.055
		n cells	15
VR	fosGFP^{-}	Mean	0.18
		SEM	0.03
		n cells	13
	fosGFP^{+}	Mean	0.169
		SEM	0.04
		n cells	12

Supplementary Table 17: GABAergic tonic current (pA/pF) - statistical comparisons

		Two-way ANOVA Source of variation	F (DFn. DFd)	p value	post-hoc Bonferroni p value
Tonic current	HC fosGFP^{-} / HC fosGFP^{+}	fosGFP	F (1. 48) = 6.803	p=0.012	** p<0.0016
	VR fosGFP^{-} / VR fosGFP^{+}	fosGFP	F (1. 48) = 6.803	p=0.012	p>0.999
	HC fosGFP^{+} / VR fosGFP^{+}	Behaviour	F (1. 48) = 4.384	p=0.041	** p=0.005
	HC fosGFP- / VR fosGFP^{-}	Behaviour	F (1. 48) = 4.384	p=0.041	P>0.999

Supplementary Table 18: sEPSC

			sEPSC Frequency (Hz)	sEPSC Amplitude (pA)	sEPSC Rise time (ms)	sEPSC Decay time (ms)	sEPSC Rise slope (pA/ms)
HC	fosGFP^{-}	Mean	0.26	6.76	0.92	4.26	7.73
		SEM	0.03	0.42	0.06	0.41	0.62
		n cells	13	13	13	13	13
	fosGFP^{+}	Mean	0.29	6.65	1.24	4.65	7.53
		SEM	0.10	0.42	0.23	0.50	1.71
		n cells	10	10	10	10	10
VR	fosGFP^{-}	Mean	0.24	7.32	1.26	5.49	6.36
		SEM	0.05	0.43	0.13	0.66	0.59
		n cells	14	14	14	14	14
	fosGFP^{+}	Mean	0.30	7.89	1.38	5.12	5.95
		SEM	0.04	0.44	0.09	0.38	0.47
		n cells	14	14	14	14	14

Supplementary Table 19: sEPSC - statistical comparisons

		Two-way ANOVA Source of variation	F (DFn. DFd)	p value	post-hoc Bonferroni p value
sEPSC frequency	HC fosGFP$^-$ / HC fosGFP$^+$	fosGFP	F (1. 47) = 0.833	p=0.3661	none
	VR fosGFP$^-$ / VR fosGFP$^+$	fosGFP	F (1. 47) = 0.833	p=0.3661	none
	HC fosGFP$^+$ / VR fosGFP$^+$	behavior	F (1. 47) = 0.0139	p=0.9065	none
	HC fosGFP$^-$ / VR fosGFP$^-$	behavior	F (1. 47) = 0.0139	p=0.9065	none
sEPSC amplitude	HC fosGFP$^-$ / HC fosGFP$^+$	fosGFP	F (1. 47) = 0.2833	p=0.5970	none
	VR fosGFP$^-$ / VR fosGFP$^+$	fosGFP	F (1. 47) = 0.2833	p=0.5970	none
	HC fosGFP$^+$ / VR fosGFP$^+$	behavior	F (1. 47) = 4.288	p=0.0439	none
	HC fosGFP$^-$ / VR fosGFP$^-$	behavior	F (1. 47) = 4.288	p=0.0439	none
sEPSC rise time	HC fosGFP$^-$ / HC fosGFP$^+$	fosGFP	F (1. 47) = 3.176	p=0.0812	none
	VR fosGFP$^-$ / VR fosGFP$^+$	fosGFP	F (1. 47) = 3.176	p=0.0812	none
	HC fosGFP$^+$ / VR fosGFP$^+$	behavior	F (1. 47) = 3.563	p=0.0653	none
	HC fosGFP$^-$ / VR fosGFP$^-$	behavior	F (1. 47) = 3.563	p=0.0653	none
sEPSC decay time	HC fosGFP$^-$ / HC fosGFP$^+$	fosGFP	F (1. 47) = 0.001	p=0.9838	none
	VR fosGFP$^-$ / VR fosGFP$^+$	fosGFP	F (1. 47) = 0.001	p=0.9838	none
	HC fosGFP$^+$ / VR fosGFP$^+$	behavior	F (1. 47) = 2.716	p=0.1060	none
	HC fosGFP$^-$ / VR fosGFP$^-$	behavior	F (1. 47) = 2.716	p=0.1060	none

sEPSC rise slope	HC fosGFP$^-$ / HC fosGFP$^+$	fosGFP	F (1, 47) = 0.1345	p=0.7155	none
	VR fosGFP$^-$ / VR fosGFP$^+$	fosGFP	F (1, 47) = 0.1345	p=0.7155	none
	HC fosGFP$^+$ / VR fosGFP$^+$	behavior	F (1, 47) = 2.983	p=0.0907	none
	HC fosGFP$^-$ / VR fosGFP$^-$	behavior	F (1, 47) = 2.983	p=0.0907	none

Supplementary Table 20: Soma size and total dendritic length

			Soma size (μm^2)	Total dendritic length (µm)
HC	fosGFP$^-$	Mean	89.52	1424.10
		SEM	9.29	94.40
		n cells	11	11
	fosGFP$^+$	Mean	93.76	1881.61
		SEM	7.52	47.14
		n cells	19	19
VR	fosGFP$^-$	Mean	88.77	1610.79
		SEM	7.99	103.64
		n cells	14	15
	fosGFP$^+$	Mean	89.24	2004.37
		SEM	6.93	116.24
		n cells	17	18

Supplementary Table 21: Soma size and total dendritic length – statistical comparisons

		Two-way ANOVA Source of variation	F (DFn. DFd)	p value	post-hoc Bonferroni p value
Soma size	HC fosGFP$^-$ / HC fosGFP$^+$	fosGFP	F (1. 57) = 0.09	p=0.7701	none
	VR fosGFP$^-$ / VR fosGFP$^+$	fosGFP	F (1. 57) = 0.09	p=0.7701	none
	HC fosGFP$^+$/ VR fosGFP$^+$	behavior	F (1. 57) = 0.11	p=0.7435	none
	HC fosGFP$^-$ /VR fosGFP$^-$	behavior	F (1. 57) = 0.11	p=0.7435	none
Total dendritic length	HC fosGFP$^-$ /HC fosGFP$^+$	fosGFP	F (1. 59) = 19.78	p<0.0001	** p=0.0038
	VR fosGFP$^-$ /VR fosGFP$^+$	fosGFP	F (1. 59) = 19.78	p<0.0001	** p=0.0072
	HC fosGFP$^+$/ VR fosGFP$^+$	behavior	F (1. 59) = 2.615	p=0.1112	none
	HC fosGFP$^-$ / VR fosGFP$^-$	behavior	F (1. 59) = 2.615	p=0.1112	none

Supplementary Figure 1. Experimental protocol in home cage and training conditions

(A) At day 1, mice underwent a surgery to chronically implant a head-bar and then were placed in their home cage (one mouse per cage). From day 2-3, they were water restricted. Some mice were left in their cage until the sacrifice (Home cage mice, HC, top), the others were trained from day 3-5, using a virtual reality's set-up (Trained mice, VR, bottom). (B) Virtual reality's set-up: mice were fixed to an arm with a screw through the chronically implanted head-bar (see

photo on the bottom right). By moving an air-flow-supported styrofoam JetBall, mice were navigating in a 270° virtual environment provided through six TFT monitors surrounding the JetBall (see the top view representation of the experimental set-up on the top right). Reward was delivered to the animal through a plastic tube (see the photo). (C) Screenshots from the middle to the right and left ends of the track; the track (150 cm) was divided into four regions with different textures (black dots, white dots, vertical green stripes and vertical black stripes). Water rewards were given at the ends of the track, with available rewards alternating between the reward sites. (D) Left panel: Sample trajectories for an individual mouse on training sessions 2 (top) and 20 (bottom); position is the animal's location along the 150 cm-long linear track axis and blue dots indicate rewards. Middle panel: Rewards frequency vs. number of sessions. Right panel: Running speed of mice as a function of the position on the linear track.

Supplementary Figure 2. Immunoreactivity of fosGFP$^+$ cells in home cage and training conditions

(A) Representative double immunostaining using anti-EGFP antibody (left panel) associated with anti-cfos antibody (middle panel) in DG of mice in home cage (HC, top) and mice trained in virtual reality (VR, bottom); the rightmost panels result from the merge of the other 2 panels; scale bar 5 µm. (B) Same as (A), with anti-Prox1 antibody (middle panel). (C) Triple immunostaining using anti-EGFP (left panel) antibody associated with anti-Prox1 (middle left panel) and anti-calretinin (CR, middle right panel) antibodies in mice trained in VR; the arrow indicates a triple immunopositive cell. The rightmost panel results from the merge of the other 3 panels; scale bar 15 µm.

Supplementary Figure3. Voltage sag in fosGFP$^+$ and fosGFP$^-$ DGCs in home cage and training conditions

(A) Representative membrane potential variations of fosGFP$^-$ (left) and fosGFP$^+$ DGCs (right) from mice in home cage (HC, top traces) and trained mice (VR, bottom traces), elicited by 500 ms hyperpolarizing current pulses decreasing by -20 pA steps until membrane potential peak value reached -120 mv (bold traces). (B) Bar graph representing the sag ratio in each condition.

Supplementary Figure 4. PTX-induced input resistance variation

Bar graph representing the PTX-induced R_{in} variation ratio (%) measured in paired conditions, i.e. in the same neuron recorded before and after PTX application.

Supplementary Figure 5. Normalized tonic GABAergic currents in fosGFP$^+$ and fosGFP$^-$ DGCs in home cage and training conditions

Bar graph representing the amplitude of tonic inhibitory current normalized by Cm in DGCs recorded in each condition.

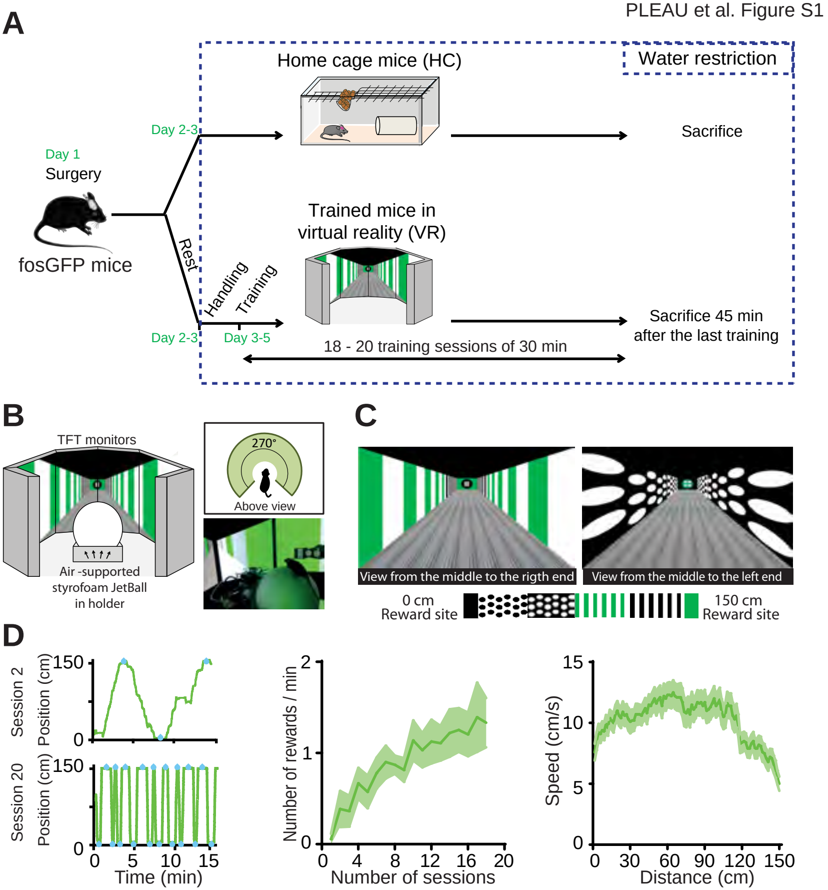
A
Home cage mice (HC)
Water restriction
Day 2-3
Sacrifice
Day 1
Surgery
fosGFP mice
Trained mice in
virtual reality (VR)
Rest
Handling
Training
Day 2-3
Day 3-5
Sacrifice 45 min
after the last training
18 - 20 training sessions of 30 min
B
TFT monitors
Air -supported
styrofoam JetBall
in holder
270°
Above view
C
View from the middle to the rigth end
View from the middle to the left end
0 cm
Reward site
150 cm
Reward site
D
Session 2
Position (cm)
Session 20
Position (cm)
Time (min)
Number of rewards / min
Number of sessions
Speed (cm/s)
Distance (cm)

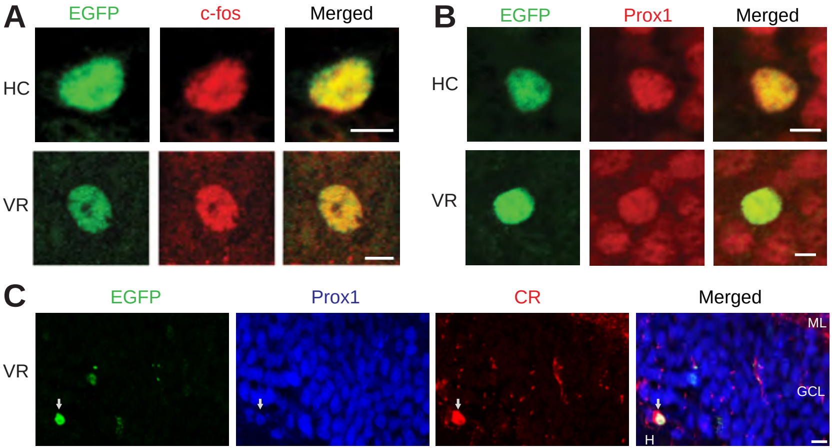
A
EGFP
c-fos
Merged
HC
VR
B
EGFP
Prox1
Merged
HC
VR
C
EGFP
Prox1
CR
Merged
VR
ML
GCL
H

PLEAU et al. Figure S3

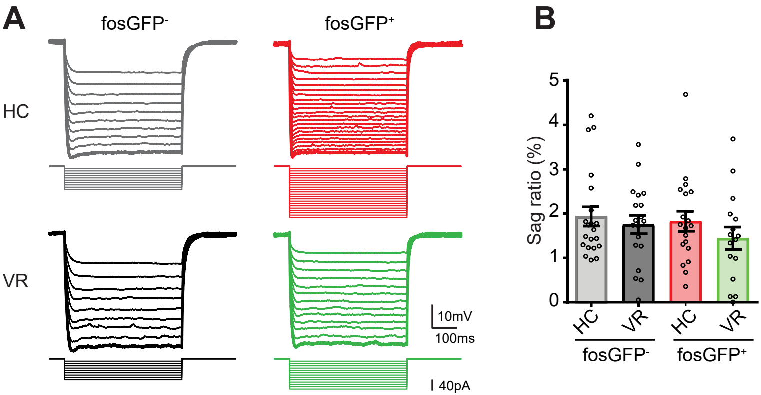

PLEAU et al. Figure S4

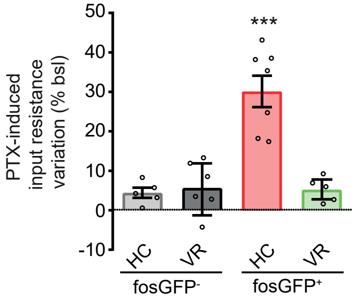

PLEAU et al. Figure S5

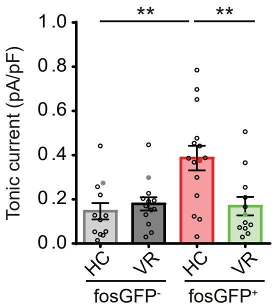

Supplement: Supplementary file 1 [file Data_Sheet_1.PDF]
